# Available Virtual Reality-Based Tools for Executive Functions: A Systematic Review

**DOI:** 10.3389/fpsyg.2022.833136

**Published:** 2022-04-11

**Authors:** Francesca Borgnis, Francesca Baglio, Elisa Pedroli, Federica Rossetto, Lidia Uccellatore, Jorge Alexandre Gaspar Oliveira, Giuseppe Riva, Pietro Cipresso

**Affiliations:** ^1^IRCCS Fondazione Don Carlo Gnocchi ONLUS, Milan, Italy; ^2^Department of Psychology, Catholic University of the Sacred Heart, Milan, Italy; ^3^Applied Technology for Neuro-Psychology Lab, Istituto Auxologico Italiano, Istituto di Ricovero e Cura a Carattere Scientifico, Milan, Italy; ^4^Faculty of Psychology, eCampus University, Milan, Italy; ^5^Universidade Lusófona de Humanidades e Tecnologias, Lisboa, Portugal; ^6^Department of Psychology, University of Turin, Turin, Italy

**Keywords:** executive functions, Virtual Reality, psychometric assessment, rehabilitation, virtual environments

## Abstract

**Introduction:**

Executive dysfunctions constitute a significant public health problem: their high impact on everyday life makes it a priority to identify early strategies for evaluating and rehabilitating these disorders in a real-life context. The ecological limitation of traditional neuropsychological tests and several difficulties in administering tests or training in real-life scenarios have paved the way to use Virtual Reality-based tools to evaluate and rehabilitate Executive Functions (EFs) in real-life.

**Objective:**

This work aims to conduct a systematic review to provide a detailed description of the VR-based tools currently developed for the evaluation and rehabilitation of EFs.

**Methods:**

We systematically searched for original manuscripts regarding VR tools and EFs by looking for titles and abstracts in the PubMed, Scopus, PsycInfo, and Web of Science databases up to November 2021 that contained the following keywords “Virtual Reality” AND “Executive function^*^.”

**Results and Conclusion:**

We analyzed 301 articles, of which 100 were included. Our work shows that available VR-based tools appear promising solutions for an ecological assessment and treatment of EFs in healthy subjects and several clinical populations.

## Introduction

“Executive function” (EF) is a complex construct, described by Chan and colleagues as “an umbrella term comprising a wide range of cognitive processes and behavioral competencies which include verbal reasoning, problem-solving, planning, sequencing, the ability to sustain attention, resistance to interference, utilization of feedback, multitasking, cognitive flexibility, and the ability to deal with the novelty” (Chan et al., 2008). Specifically, these higher-order cognitive abilities and behavioral skills are responsible for controlling and regulating actions (e.g., starting and stopping activities or monitoring) (Burgess and Simons, 2005; Chan et al., 2008) and performing complex or non-routine tasks (e.g., ability to perform two tasks simultaneously) (Godefroy, 2003; Alvarez and Emory, 2006; Alderman, 2013). Several studies have shown the critical role of executive functioning in performing various activities of daily living (ADL) (Fortin et al., 2003) and especially the instrumental activities of daily living (IADL), such as preparing meals, managing money, shopping, doing housework, and using a telephone (Chevignard et al., 2000; Fortin et al., 2003; Vaughan and Giovanello, 2010). Due to this overt role in everyday functioning, the executive impairment, known as “Dysexecutive Syndrome” (Robertson et al., 1997; Snyder et al., 2015), has a relevant impact on personal independence, ability to work, educational success, social relationships and cognitive and psychological development (Green, 1996; Goel et al., 1997; Green et al., 2000), with consequences on a person's quality of life and feelings of personal wellbeing (Gitlin et al., 2001). In recent years, the cognitive neuroscience of EFs has been rapidly developing, driven by technological progress, which claimed the crucial role of the frontal lobe in supporting executive processes involved in many real-life situations (Burgess et al., 2006). Dysexecutive Syndrome appears to be associated with aging of the prefrontal cortex in the healthy elderly population (Raz, 2000; Burke and Barnes, 2006), but also is typical in neurological or psychiatric patients due to frontal lobe damage, such as after traumatic brain injury (TBI) and stroke (Baddeley and Wilson, 1988; Nys et al., 2007) or specific pathologies such as Parkinson's disease (PD) (Aarsland et al., 2005; Kudlicka et al., 2011) and Multiple Sclerosis (MS) (Nebel et al., 2007). However, EFs' impairments can be linked to other cerebral areas due to the connection of frontal regions with cortical and subcortical areas, such as the amygdala, cerebellum, and basal ganglia (Tekin and Cummings, 2002).

Since EFs have adverse effects in performing activities of daily living (Fortin et al., 2003; Vaughan and Giovanello, 2010), the identification of early strategies functional to the evaluation and rehabilitation of EFs are critical to minimize the effects of these executive impairments and improve everyday function (Levine et al., 2007). However, the assessment and rehabilitation of EFs represent a challenge due not only to the complexity and heterogeneity of the construct (Stuss and Alexander, 2000) but also to methodological difficulties (Goldstein, 1996; Chaytor and Schmitter-Edgecombe, 2003; Barker et al., 2004; Crawford and Henry, 2005; Godefroy et al., 2010; Kudlicka et al., 2011; Serino et al., 2014).

As regards the evaluation, EFs are traditionally assessed with laboratory tasks or paper-and-pencil neuropsychological tests based on the theory, such as the Modified Wisconsin Card Sorting Test (WCST) (Nelson, 1976) or the Trail Making Test (TMT) (Reitan, 1992a), which guarantee standardized procedures and scores. Over the years, an increasing number of tests have been developed to assess different patients (Chan et al., 2008). The assessment protocol may include a single task for the evaluation of a single cognitive process, for example, Tower of London (ToL) for problem-solving abilities (Allamanno et al., 1987) or tests batteries to assess the entire executive functioning, such as the Frontal Assessment Battery (FAB) (Dubois et al., 2000; Appollonio et al., 2005). However, several authors have shown many limitations and disadvantages in the traditional neuropsychological evaluation (Schultheis and Rizzo, 2001; Parsons and Rizzo, 2008). Firstly, traditional paper and pencil tests could present reliability problems (Rizzo et al., 2001) since the tests could negatively be affected by the different administration procedures (e.g., examiners, test environment, quality of the stimuli or scoring errors). Therefore, validated computerized versions of traditional neuropsychological tests were developed, offering the advantage of systematically delivering stimuli and the ability to monitor speed and accuracy with precision. However, even these versions do not detect how cognitive functioning can change in stressful everyday situations (Armstrong et al., 2013). Secondly, several studies revealed that many patients with Dysexecutive Syndrome achieve normal scores on traditional neuropsychological tests and, at the same time, complain of substantial difficulties in daily life activities (Shallice and Burgess, 1991). This problem may result from a lack of ecological validity of the tests for EFs (Chan et al., 2008). A test can be defined ecological if (1) the task corresponds, in form and content, to a situation outside the laboratory (representativeness of the task), and (2) a poor performance on the test is predictive of problems in the real world (generalizability of the results) (Kvavilashvili and Ellis, 2004). Traditional paper and pencil tests require simple responses to a single event, while complex everyday tasks may require a more complex set of responses (Chan et al., 2008). In other words, the situation - usually the clinic - in which patients perform the tests is different from most of the conditions encountered outside it (that is, they show little “representativeness”). Therefore, the traditional assessment appears not to be able to predict the complexity of executive functioning in real-life settings reliably (Shallice and Burgess, 1991; Goldstein, 1996; Klinger et al., 2004; Burgess et al., 2006; Chaytor et al., 2006; Chan et al., 2008). Nevertheless, an ecological assessment is crucial to understand how cognitive deficits (above all EF) affect daily functioning (Manchester et al., 2004; Burgess et al., 2006). In other words, it allows evaluating if patients can effectively manage and orient cognitive resources within the complexity of the external world (Crawford, 1998; Rand et al., 2009). Since EFs play a key role in everyday life (Shallice and Burgess, 1991) and independent functioning, it is necessary that the EF clinical tests have ecological validity. In this framework, Burgess and colleagues proposed neuropsychological assessments based on models derived from directly observable daily behaviors (Burgess et al., 2006). This “function-led” approach differs from the emphasis on abstract cognitive “constructs” by paying attention to the role of EFs within the complexity of the “functional” behaviors found in real-life situations. This innovative approach could lead to tasks more suited to the clinical concerns due to the transparency offered by greater “representativeness” and “generalizability.” In conclusion, an ecological assessment allows a deeper comprehension of the neuropsychological profile of the patient and future personalized (Pedroli et al., 2016). To overcome this ecological issue, clinicians and researchers paid attention to develop tests able to evaluate the different components of executive functioning in real-life scenarios (Chaytor and Schmitter-Edgecombe, 2003; Jurado and Rosselli, 2007), such as the Multiple Errands Test (MET) (Shallice and Burgess, 1991; Alderman et al., 2003) and Behavioral Assessment of the Dysexecutive Syndrome (BADS) (Wilson et al., 1997). Specifically, MET is a functional test requiring simple tasks (e.g., buying six items) in a real supermarket. At the same time, BADS is a laboratory-based battery that includes ecological tasks (e.g., temporal judgement, rule shift cards, action program, key search) and a dysexecutive questionnaire that investigates several domains like personality, motivation, behavioral, and cognitive changes. The assessment of EFs in real-life settings provides a more accurate estimate of the patient's deficits than within laboratory conditions (Rand et al., 2009) but showed further limitations, such as long times, high economic costs, the difficulty of the organization (e.g., requests for authorisations from local companies), poor controllability of experimental condition or applicability with patients with significant behavioral, psychiatric and motor difficulties (Bailey et al., 2010).

The ecological limitations of traditional neuropsychological tests and several difficulties in administering tests in real-life scenarios have paved the way to use technological tools such as Virtual Reality (VR) to assess EFs in real life (Bohil et al., 2011). VR is a sort of human-computer interface system that enables designing and creating realistic spatial and temporal scenarios, situations or objects that, reproducing conditions of daily life, could allow an ecologically valid evaluation of EFs (Lombard and Ditton, 1997; Campbell et al., 2009; Bohil et al., 2011; Parsons et al., 2011; Parsons, 2015). Therefore, VR could facilitate the assessment and rehabilitation of possible impairments in individuals with executive dysfunction (Tarnanas et al., 2013), leading clinicians to observe in real-time their patients in an everyday setting. These Virtual Environments (VEs) enable patients to interact dynamically with computer-simulated objects and 3D settings (Pratt et al., 1995; Climent et al., 2010) that could allow reproducing complex emotional and cognitive experiences (such as planning and organizing practical actions, attention shift) resembling everyday life situations (Castelnuovo et al., 2003) in ecologically valid and controlled environments. Overall, VR could allow evaluating everyday difficulties due to executive dysfunctions and train these impairments, working directly on impaired ADL and IADL (Zhang et al., 2003; Klinger et al., 2006).

In addition to allowing an ecological assessment, VR-based tools appear highly flexible and guarantee simultaneously a controlled and precise presentation of a large variety of stimuli (Armstrong et al., 2013) and the collection of the full range of users' answers that can be objectively measured (Rizzo et al., 2001; Parsons et al., 2011; Parsons, 2015). Therefore, VR could integrate traditional neuropsychological assessment procedures and improve their reliability and psychometric validity (Riva, 1997, 2004; Rizzo et al., 2001). Moreover, VR can recognize and monitor facial expressions and body movements: all gestures could be captured and processed by translating them into other actions (e.g., grasping, virtual environment scrolling or dropping objects, blowing and moving elements) that manage the virtual objects' direct manipulation using natural behavior (Parsons et al., 2011).

In a rehabilitative context, VR also shows other valuable advantages, showing itself a promising tool in training ADLs' skills (Zhang et al., 2003; Klinger et al., 2006). Firstly, it allows individualized treatment according to patients' skills and needs (Lo Priore et al., 2002; Rand et al., 2009): the real-time data acquisition and performance analysis (Parsons et al., 2011; Parsons, 2015) guarantee the possibility of customizing the scenarios in real-time, focusing on the patient's characteristics and demands (Castelnuovo et al., 2003; Rand et al., 2009). Moreover, VR-based tool also allows compensation for sensory deprivation and motor impairments through multisensorial stimulation and feedback (Kizony, 2011; Zell et al., 2013; Nir-Hadad et al., 2017). Indeed, VR allows administering stimuli and instructions through different modalities (visual, auditory, tactile), which can be adapted to possible sensory deficits of the patients (Parsons and Rizzo, 2008). Another strength of VEs concerns presenting scenarios with features not available in the real world (Kizony, 2011; Zell et al., 2013; Nir-Hadad et al., 2017): cueing stimuli provided to patients to help them in compensatory strategies, to improve functional behavior day by day (Rizzo et al., 2001). Furthermore, VR enables them to perform exercises at a distance, in the comfort and safety of their homes (Dores et al., 2012). This result has been relevant since it makes it possible to overcome two crucial clinical issues: long waiting lists of health services and difficulties in moving patients between their homes and health services. Moreover, the rehabilitation with VR appeared cheaper than the traditional one since, for example, it allows to recreate complex everyday scenarios (e.g., the presence of more persons at the same time), avoiding the need to leave the rehabilitation setting. Finally, VR allows for gradually increasing tasks' complexity, maintaining experimental control over stimulus delivery and individualizing treatment needs in a standardized manner (Rand et al., 2009). Overall, several studies converge that VR is a promising tool to improve rehabilitation since it allows the provision of meaningful, versatile and individualized tasks that can enhance patients' motivation, enjoyment and engagement during training (Hayre et al., 2020), overcoming scarce compliance of patients with cognitive dysfunctions about the traditional rehabilitating program, usually repetitive and not stimulating (Castelnuovo et al., 2003; Rand et al., 2009). Interestingly, several studies have shown that the VR tools' realism and engagement could help transfer learning to the real world (Klinger et al., 2004; Rizzo and Kim, 2005; Carelli et al., 2008).

In light of these promising premises, this review aims to provide a detailed description of the VR tools currently developed for the evaluation and rehabilitation of EFs.

## Methods

We achieved this systematic review agreeing to the Preferred Reporting Items for Systematic Reviews and Meta-Analyses (PRISMA) guidelines and flow diagram (Liberati et al., 2009).

### Information Sources and Study Selection

The literature was searched in the electronic databases PubMed, Web of Science, Scopus and PsycInfo from inception to November 2021. Bibliographies identified articles, and a manual search of relevant journals for additional references was conducted. A further search on Google Scholar and the bibliography of previous reviews was also done. We used the keywords “Virtual Reality” AND “Executive function^*^” (the asterisk indicates that the search term was not limited to that word). Two reviewers (FB; CP) independently conducted the data extraction.

### Eligibility Criteria

**Figure 1:**
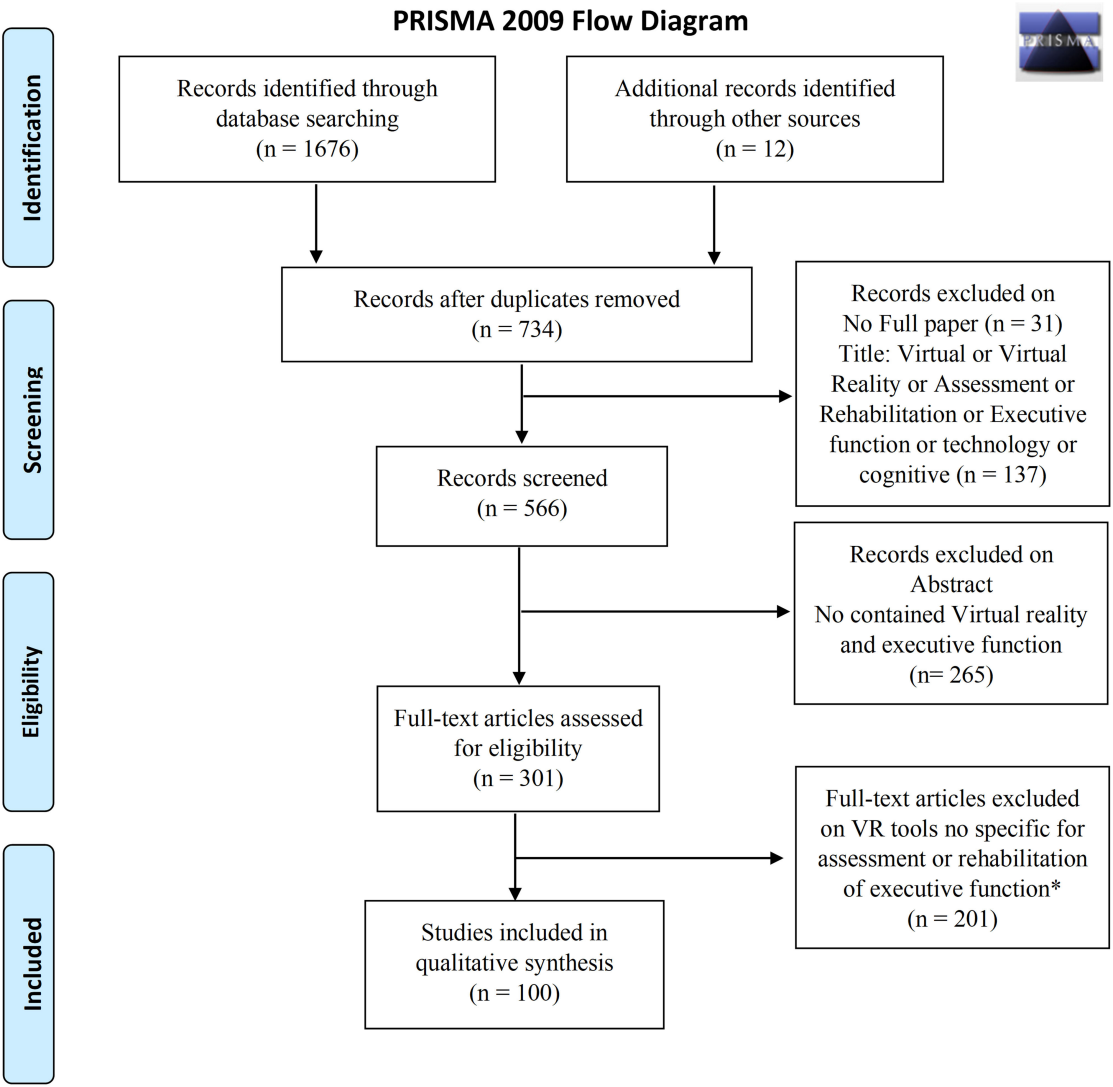
PRISMA 2009 flow diagram.

Studies were included if they fulfilled the following criteria: Virtual Reality-based tools specific for the assessment or rehabilitation of EFs. Exclusion criteria were no full paper (i.e., books, chapters of the books, qualitative studies, letters, comments, dissemination, published abstracts without text) and non-English language. The selection of studies was first based on screening the title and abstract, followed by reading the full text of the remaining reports (Figure 1).

## Virtual Reality Tool

**Figure 2:**
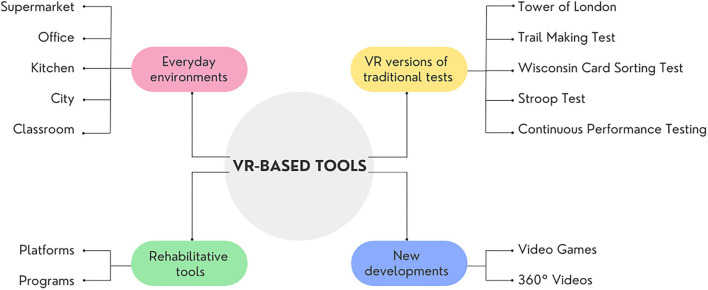
Overview of available VR-based tools.

This review aims to provide a detailed description of the main tools that exploit VR for the assessment and rehabilitation of EFs. The description of the tools has been organized into paragraphs based on the VEs used (e.g., supermarket, kitchen). Further paragraphs have been introduced to offer an overview of the main platforms and programs used to evaluate and rehabilitate EFs and the virtual reality versions of traditional paper-pencil tests. Finally, we have decided to introduce two sections to describe the development of Games and 360° videos as innovative and feasible solutions for the assessment and rehabilitation of EFs (Figure 2).

For each VR-based instrument reviewed, we have provided a complete description of the tool and, if available, information about usability, construct validity, discriminant validity and test re-test reliability (for a summary, see Table 1).

**Table 1:** Summary of available psychometric proprieties of VR-based assessment tools.

	**Usability**	**Construct validity/convergent validity**	**Discriminant validity/efficacy in discriminating between populations**	**Test-retest reliability**
**Everyday environments:**
Virtual Errands Test	YES	YES	HC vs. PD/Stroke HC vs. OCD/Schizophrenia	NO
Virtual Environment Grocery Store	NO	YES	NO	NO
Adapted Four-Item Shopping Task	YES	YES	HC vs. Stroke	NO
Virtual Action Planning - Supermarket	NO	YES	HC vs. MCI/Stroke/Schizophrenia	NO
Virtual Supermarket Shopping Task	NO	NO	NO	NO
Jansari Assessment of Executive Functions	NO	YES	HC vs. ABI	NO
Jansari Assessment of Executive Functions for Children	NO	NO	NO	NO
Assessim Office	NO	YES	HC vs. TBI/MS	NO
EcoKitchen	NO	NO	HC vs. HD manifest/premanifest	NO
VR-cooking task	NO	YES	HC vs. Alcohol Use Disorder	NO
Kitchen and cooking	NO (acceptability)	NO	NO	NO
Multitasking in the City Test	NO (acceptability)	YES	HC vs. ABI	NO
Virtual Library Environment	NO	YES	HC vs. TBI	NO
Edinburgh Virtual Errands Test	NO	YES	NO	NO
Virtual Reality Day-Out Task	NO	NO	HC vs. MCI vs. AD	NO
Virtual Classroom	NO	YES	HC vs. children with ABI/ NF1/ADHD	NO
**Virtual versions of traditional paper and pencil tests:**
Tower of London	NO	YES	NO	NO
Virtual Reality Color Trails Test	NO	YES	NO	YES
Look for a Match	NO	YES	NO	NO
Virtual Reality Stroop Task	NO	YES	NO	NO
The Virtual Classroom Stroop Task	NO	YES	NO	NO
The Virtual Apartment Stroop Task	NO	YES	NO	NO
Virtual Classroom Bimodal Stroop	NO	YES	HC vs. Autism Disorders	NO
Virtual Reality Continuous Performance Testing	NO	YES	HC vs. ADHD	NO
Advanced Virtual Reality Tool for the Assessment of Attention	NO	NO	NO	NO
Nesplora Aquarium	YES	YES	HC vs. ADHD low vs. elevated Mood Disorders	NO
**New developments:**
Virtual Reality Video Game	NO	YES	NO	NO
Virtual Reality avatar interaction platform	NO (engagement)	NO	Personnel military with/without TBI	NO
Picture Interpretation Test 360°	NO	YES	HC vs. PD/MS	NO
EXecutive-functions Innovative Tool 360°	YES	YES	NO	NO

*HC, Healthy Controls; PD, Parkinson's Disease; MS, Multiple Sclerosis; TBI, Traumatic Brain Injury; ADHD, Attention-Deficit/Hyperactivity Disorder; ABI, Acquired Brain Injury; OCD, Obsessive and Compulsive Disorders; MCI, Mild Cognitive Impairments; HD, Huntington's disease; NF1, Neurofibromatosis type 1*.

### Virtual Supermarket Environment

In the literature, many studies have focused on developing virtual shopping environments that simulate a real supermarket to evaluate and treat EFs (Nir-Hadad et al., 2017). Shopping has been selected as an activity that characterizes the IADL, essential for everyday life. Indeed, this activity includes tasks/actions that require the use of EFs, such as comprehending a store's design, forming a strategy to identify the location of products of different types and costs, differentiating between products and keeping track of products acquired. Specifically, most studies have focused on developing and testing the virtual version of the Multiple Errands Test (VMET).

#### Virtual Multiple Errands Test

VMET is a complex shopping task in which the participants must carry out different tasks in compliance with various rules. Two virtual scenarios of the V-MET have been created: the IREX V-Mall supermarket (Rand et al., 2005, 2009) and the NeuroVR supermarket (Raspelli et al., 2009; Riva et al., 2009).

Rand and colleagues have developed a first version of the VMET (Rand et al., 2009), set in the V-Mall (Rand et al., 2005), a virtual supermarket programmed by GestureTek's Interactive Rehabilitation and Exercise System (IREX) video-capture VR system. Participants can interact with the VE through arrows and natural arm movements (e.g., touch products with both hands). VMall simulates a real supermarket with different stores and aisles: each aisle consists of a maximum of 60 products arranged on the shelves and divided into different categories (e.g., bakery products, cleaning items). The products are reproductions of photographs of real items, taken with a digital camera and rendered using 3D graphic software. The therapist can select and order products: the number, type, and position of objects on the shelves can vary in each corridor. The authors added some common features such as background music or typical special sales announcements to improve the sense of immersion. In their work, Rand and colleagues have provided initial support for the ecological validity of the VMET as an assessment tool of EFs. The preliminary results showed that VMET is sensitive to brain injury because it was able to differentiate between healthy control subjects and patients with post-stroke (Rand et al., 2009).

Raspelli et al. have developed another VR-based MET using the VR platform NeuroVR software (Raspelli et al., 2009, 2010; Riva et al., 2009; Wiederhold et al., 2010). Thanks to this platform, Raspelli and colleagues created a new scenario for assessing EFs (Raspelli et al., 2009). The original procedure of the MET (Shallice and Burgess, 1991) was modified to be adapted to the virtual scenario of the supermarket (Pedroli et al., 2013). In general, the VMET consists of a Blender-based application that allows the assessment of different aspects of EFs through active exploration of a virtual supermarket, where participants must select and buy various products arranged on shelves, following a predefined list obtain some information and respect different rules. Precisely, the VMET measures a subject's ability to formulate, store, and check all the goals and subgoals to respond to environmental demands in ecological situations and to complete specified tasks. In this way, the EFs stimulated are multiple, from the ability to plan a sequence of actions to problem-solving and to cognitive and behavioural flexibility (Cipresso et al., 2013b). Within the virtual supermarket, the products are grouped into the main categories of foods, such as drinks, fruit and vegetables, breakfast foods, hygiene products, frozen foods and products for gardens and pets. Moreover, some signs indicating the product categories have been inserted in the upper part of each section to help the subjects in their exploration (Raspelli et al., 2009; Cipresso et al., 2014). The VMET is composed of four main tasks: 1) buying six different products (i.e., one product on sale); 2) requiring the examiner information about one product to acquire; 3) writing the shopping list of products bought after 5 min from the beginning of the test; 4) answering some questions at the end of the virtual session (i.e., which is the closing time of the supermarket? how many shelves sell the fruit? how many departments are there in the supermarket?) (Cipresso et al., 2013a, 2014). To complete the tasks, participants must follow eight rules: (1) performing all the proposed tasks; (2) performing all the tasks in any order; (3) not going to a place if it is not part of a task; (4) not going to the same passage twice; (5) not acquiring more than two products for each category; (6) completing the exercise in the shortest possible time; (7) not talking to the experimenter if it is not part of a task; and (8) going to “shopping cart” and making a list of all their products, after 5 min from the beginning of the task (Raspelli et al., 2009).

Before starting the real task, the participants perform an initial training phase in a smaller supermarket to test the joy pad use (Pedroli et al., 2013) and understand how to move in the environment (Pedroli et al., 2016). In this phase, the subjects explore the VE freely for a few minutes or until they learn the use of the joypad. After training, the examiner shows the new virtual supermarket, describing different sections and giving a shopping list, a map of the supermarket, information about the supermarket (i.e. opening and closing times, products on sale), a pen, a wristwatch and the instruction sheet. Moreover, the examiner reads and explains all the instructions to the subject to guarantee complete understanding (Cipresso et al., 2014). After that, the participant can freely navigate in the virtual supermarket using a joypad (with the arrows “up-down” joystick) and collect products (by pushing a button on the right side of the joypad) (Raspelli et al., 2012; Cipresso et al., 2013b; Pedroli et al., 2016). The examiner cannot speak to subjects during the task or answer the questions. Still, he can only take notes on the participant's behaviours in the VE (Pedroli et al., 2016) and execution time: clinician measured the time, stopping it when the subject says “I finished” (Raspelli et al., 2009; Cipresso et al., 2013a). In order to better understand the subject's performance, five different items must be registered: total errors (task failure), inefficiencies, strategies, rule breaks, interpretation failures (Shallice and Burgess, 1991; Raspelli et al., 2012). Specifically: 1) errors or task failure: a task is not correctly completed. The total score ranges from 11 (participants complete all task correctly as indicated by the test) to 33 (participants complete all tasks incorrectly). 2) inefficiencies: the participants could have used a more effective strategy to complete the task (i.e., not grouping similar tasks when possible). The general scoring range is 8 (many inefficiencies) to 32 (none). 3) strategies; to analyse their ability to use strategies, 13 behaviours that facilitated carrying out the tasks are evaluated (i.e., accurate planning before starting a specific subtask). The total score ranges from 13 (good strategies) to 52 (no strategies). 4) rule breaks: The total score ranges from 8 (many rule breaks) to 32 (no rule breaks). Notably, the scoring scale for each inefficiency/strategy/rule break ranged from 1 to 4 (1 = always; 2 = more than once; 3 = once; 4 = never). 5) interpretation failures: the requirements of a particular task are misunderstood (i.e., subjects think that the subtasks must be performed in the order of presentation in the information sheet). The score for each interpretation failure ranges from 1 to 2 (1 = yes; 2 = no); therefore, the general score ranges from 3 (a large number of interpretation failures) to 6 (no interpretation failures). (Raspelli et al., 2009).

Furthermore, for every subtask, other variables can be analysed (partial tasks failures): 1) sustained attention (not distracted by other stimuli); 2) maintaining the correct sequence of the task; 3) searched item in the correct area; 4) maintained task objective to completion; 5) divided attention between components of task and components of other VMET task; 6) correct organisation of the materials during all task; 7) self-corrections; 8) absence of perseverations. The general score ranges from 8 (no errors) to 16 (many errors), while a scoring range for each item from 1 (yes) to 2 (no) (Raspelli et al., 2009, 2012; Pedroli et al., 2019). The VMET has demonstrated good inter-rater reliability, showing an intraclass correlation coefficient (ICC) of 0.88 (Cipresso et al., 2013b) and good usability (i.e., this test can be used with patients who are not familiar with computerized tests) (Pedroli et al., 2013). To evaluate the reliability of the VMET, the researchers have conducted two different experiments that showed that the test has good reliability: in the first, two independent researchers analyzed 11 videos in which 11 healthy subjects were tested with VMET; in the second one, seven researchers scored two videos of 2 healthy subjects running the VMET. Moreover, to analyse the usability of VMET, Pedroli and colleagues used the System Usability Scale [SUS, (Brooke, 1996)] in a sample of 21 healthy participants and 3 patients with PD. Results showed good usability of VMET for healthy subjects and that a good training phase before the test is crucial to apply the virtual protocol to PD patients (Pedroli et al., 2013).

Finally, VMET appeared sensitive to assess several components of EFs in neurological and psychiatric populations (Wiederhold et al., 2010; Raspelli et al., 2012; Cipresso et al., 2013a; Pedroli et al., 2019), offering an accurate evaluation of deficits hardly detectable with traditional tests (Cipresso et al., 2014). As regards the neurological condition, the studies have focused on the feasibility of VMET as an assessment tool of EFs in PD and post-stroke patients, showing promising results in terms of convergent validity (good correlation between VMET scores and traditional paper-and-pencil tests, such as ToL, FAB and TMT) and efficacy in distinguishing between healthy controls and pathological groups (Raspelli et al., 2009; Albani et al., 2010). Taking up these research, Cipresso and co-workers deepened the validity of VMET in PD with normal cognition populations (Cipresso et al., 2014), showing significant differences in the VMET scores but not in traditional tests between PD patients and control subjects, particularly in cognitive flexibility. This study offers preliminary evidence that a more ecologically valid evaluation of EFs is more likely to early detect subtle executive deficits in PD patients (Cipresso et al., 2014). Regarding the psychiatric population, La Paglia and colleagues successfully conducted three studies evaluating the feasibility of VMET as an assessment tool of EFs in patients with Obsessive-Compulsive disease (OCD) and schizophrenia. Results showed a good convergent validity of VMET and its ability to distinguish between healthy controls and both OCD and schizophrenia populations (in planning, mental flexibility and attention) (La Paglia et al., 2014). Recently, Pedroli and colleagues proposed successfully a computational approach based on classification learning algorithms to discriminate OCD patients from a control group (Pedroli et al., 2019). This good result opens a new scenario for future assessment protocols based on VR and computational techniques that could reduce time and effort for both patients and clinicians, allowing more personalized and efficient rehabilitative treatment.

Overall, VMET allows the possibility to assess some subcomponents of executive functions in ecologically valid settings, giving an accurate analysis of patients' deficits as well as traditional tests. Further study will have to analyze the temporal stability of VMET, namely test-retest reliability and criterion validity.

#### Virtual Environment Grocery Store

The Virtual Environment Grocery Store (VEGS) is another task built on MET (Law et al., 2006). The VEGS is a 3D virtual grocery store environment developed to assess executive abilities (Parsons et al., 2008). VEGS was developed using the NeuroVR platform to offer an immersive VR version of the MET, in which participants interact with avatars and objects to perform various shopping tasks (Parsons and McMahan, 2017; Parsons et al., 2017). The different shopping commissions must be completed in a VE according to some rules, in low and high distraction conditions (Shallice and Burgess, 1991; Parsons et al., 2008).

The VEGS puts the subject in an immersive modality, in which the VE is displayed using a desktop monitor. The subjects interact with the VE using the keyboard arrows and a mouse. In the VEGS, participants navigate the store and perform various tasks, such as navigating to the pharmacy and dropping off a prescription with a virtual pharmacist. Here the participant receives a number and listens for that number, ignoring other numbers and announcements while shopping. The participants must also buy products on the shopping list. When they hear their number over the public-address system, they must return to the pharmacist to pick up their prescription (event-based prospective memory). Additional tasks include: 1) navigate the virtual grocery store following specific routes through the aisles; 2) find and select the ingredients necessary for the preparation of easy eats (i.e., making peanut butter); 3) ignore products that are not on the shopping list; 4) selection of products so you don't spend more than the expected amount; 4) perform a prospective memory task when a specific individual is met (Parsons et al., 2008). Also, the difficulty of the tasks increases through the addition of distractions: 1) growing number of items to store; 2) adding background music; 3) increasing its loudness (i.e., an announcement of commercial promotions, human laughter, coughing, falling goods, crying children and ringtones of cell phones); 4) adding virtual human avatars that walk in the environment or are lined up at the control desk and in the pharmacy. Other avatars speak in small groups or on virtual phones (Parsons et al., 2017). After the VEGS, the participant performs delayed free and cued recall of the VEGS shopping items.

A preliminary study conducted on healthy university students showed the absence of correlation between VEGS and DKEFS Color-Word Interference, a traditional neuropsychology test of executive functioning. However, a second study demonstrated that the addition of environmental distractors into VEGS might be successfully used in situations where the neuropsychologist is interested in looking at both memory and inhibitory control in a distracting environment.

Within the line of research on developing virtual shopping environments that simulate the supermarket environment, some researchers have focused on adapting the Four-Item Shopping Task, an assessment IADL of shopping.

#### Adapted Four-Item Shopping Task

Some studies have validated two versions of the Adapted Four-Item Shopping Task, in which participants have to perform the shopping task in a virtual shopping environment (Kizony et al., 2017; Nir-Hadad et al., 2017). The first one was based on the original task (Rand et al., 2007), where the participant must acquire four different products that appear on a shopping list and are located in two different aisles on both the top and middle shelves. In the other version, the subject must buy four additional products that appeared on a shopping list from at least two different stores (Kizony et al., 2017). While shopping, the subjects need to consider the product brand (some brands are more expensive than others) and acquire all four items without exceeding the specified budget.

The Adapted Four-Item Shopping Task of Nir-Hadad and colleagues was performed in Virtual Interactive Shopper (VIS), a SeeMe supported virtual mall shopping environment (Hadad et al., 2012). SeeMe is a camera tracking VR system installed on any portable computer and displayed on any standard TV monitor. Currently, the virtual mall shopping environment includes three different stores: a supermarket, a toy store, and a hardware store. The types (i.e., products from a specific country), quantities, and position of the products in each store can be easily regulated. The participant navigates within and between the shopping aisles by “touching” directional arrows and selects the desired items by “hovering” over photos of the products. When a product is touched, its name is voiced. After the selection, the product's image is placed in a virtual shopping cart. The shopping list and the contents of the cart (i.e. the products already acquired) can be viewed at any time by “touching” the menu icon. Products bought by mistake can be removed from the cart. After completing the task, a detailed report of the shopping activity is generated, including information about products selected (what and when), if the products purchased by mistake were returned, the total cost of the acquired items and distance traversed shopping. In particular, the last variable, “distance traversed,” refers to the distance moved by participants while they were making their purchases in the virtual supermarket.

The Adapted Four-Item Shopping Task of Kizony and colleagues was performed in another shopping mall: EnvironSim Virtual Shopping Mall (Kizony et al., 2003, 2017). The Center One mall, a real shopping mall in Jerusalem, was simulated using EnvironSim software that allows personalizing any shopping mall design, including the number and type of stores and the products purchased in each store. The participant's point of view is first-hand, so the subjects can observe the environment as if they were in the real world. Distance travelled, trajectory, visited stores, products acquired, and budget management are recorded and used to calculate variables. To navigate right, left, forward and backwards, the participants must use the keyboard keys: it is also possible to replay the route taken by the shopper. The participant must use the mouse to interact with the simulation program's menu items, shopping list and shopping cart. In each shop, the images, names and prices of the products are displayed on the screen. The shopping list with category names of products to buy and the amount of money given to the subjects are located on the left. On the opposite side, there is the shopping cart with the purchased items and their prices. The participant can return the products by selecting the trash image above the object.

In both versions of the Adapted Four-Item Shopping Task, the outcomes measure included: 1) the time to acquire/select the first item, 2) total time to buy the four items, 3) a number of errors (missing items, extra items, items purchased by mistake), 4) discrepancies between the amount of money that participants could spend and the actual amount that they spent, 5) distance travelled while shopping, 6) cognitive strategies used during shopping.

The validation studies of the Adapted Four-Item Shopping Task involving healthy controls subjects and post-stroke patients have provided good convergent validity and efficacy results. Specifically, Nir-Hadad and colleagues have shown this VR-based tool's ability to differentiate healthy and pathological groups in performing executive tasks (with clinical groups that obtained lower performance). Moreover, both research teams showed correlations between performance in the Four-Item Shopping Task in the VE and clinical assessments of EFs for healthy and pathological samples (i.e., TMT, BADS and Executive Function Performance Test), indicative of a good convergent validity. In addition, Kizony and colleagues showed that their version of the test could evaluate age-related EFs decline in terms of inhibition and processing speed in healthy older adults, compared to young adults (Kizony et al., 2017). Interestingly, both healthy groups gave positive reports regarding their VE experience, but the older adults reported a lower level of usability.

#### Virtual Action Planning - Supermarket

Another user-friendly VR-based tool designed to evaluate and train the ability to plan and perform a shopping task (Klinger et al., 2004) is the Virtual Action Planning - Supermarket (VAP-S) (Klinger et al., 2006). The original VAP-S was adapted by Klinger for use by an Israeli population; the names of the aisles and grocery items and all the task elements were translated to Hebrew (Josman et al., 2006). The VAP-S simulates a fully textured, medium-size supermarket with multiple aisles displaying most of the products that can be found in a real supermarket (i.e., drinks, canned food, fruit, salted and sweet food, cleaning equipment, clothes and flowers). In this virtual supermarket, many elements were introduced: four cashier check-out counters, a reception point and a shopping cart. It also contains refrigerators for milk and dairy products, freezers, four specific stalls for fruits, vegetables, meat, fish, and bread. Moreover, some obstacles (i.e., packs of bottles) were placed to hinder the shopper's progress along the aisles. In addition, static virtual humans, such as a fishmonger, a butcher, check-out cashiers and some customers, populated the supermarket (Josman et al., 2006, 2009). Before starting the task, participants perform a training task similar to the test to familiarize subjects with the VE and the tools. During the training, some instructions are provided on the screen, and the examiner explains other general information about the task and the use of VAP-S. The individual must sit (or stand) in front of a laptop monitor and interact with the VE using a mouse and computer keyboard (Aubin et al., 2018). The participants enter the supermarket behind the cart as if they are pushing it and navigating freely by pressing the keyboard arrows. They experience the VE from a first-person perspective without any intermediating avatar. The participants must acquire seven products from a list of products, then proceed to the cashier's desk, and pay for them. Twelve correct actions (e.g., selecting the exact product) are required to complete the task correctly. The list of products is displayed on the right-hand side of the screen. The participant can select items by pressing the left mouse button. If the item chosen belongs to the list, it will be automatically transferred to the cart. Otherwise, the product will not move, and a mistake will be recorded. At the cashier check-out counter, the participant must place the items on the conveyor belt by pressing the left mouse button with the cursor pointing to the belt. He may also return an item placed on the conveyor belt to the cart. The patient can pay and proceed to the supermarket exit by clicking on the purse icon. The task is completed when the subjects leave the supermarket with the cart (Aubin et al., 2018). The VAP-S records various outcome measures (positions, times, actions) while the participant explores the VE and executes the task. Eight variables are calculated from the recorded data: 1) total distance traversed in meters, 2) whole task time in seconds, 3) number of items acquired, 4) a number of correct actions (i.e. selecting the exact product), 5) number of incorrect actions, 6) number and combined duration of pauses, 7) time to pay (i.e., the time between when the cost is displayed on the screen and when the participant clicks on the purse icon). The participants can make many errors: 1) chooses wrong items or the same item twice; 2) selects a check-out counter without any cashier; 3) leaves the supermarket without purchasing anything or without paying; or 4) stays in the supermarket after the purchase (Josman et al., 2008, 2009; Cogné et al., 2018). The eight outcomes can be conceptualized in terms of executive functioning into two categories: 1) “task completion” measured by the number of purchased products and correct actions; 2) “efficiency” that is competency in performance or ability to complete work with minimum expenditure of time and effort, measured by time, distance, and incorrect actions (Josman et al., 2009). To summarize, the main EF components are measured by looking at the participants' planning abilities within the VAP-S and their organization in time and space (Werner et al., 2009).

As a VR platform, the VAP-S appeared a valid and reliable method to assess EF disabilities in neurologic (i.e., post-stroke and mild cognitive impairment) and psychiatric (i.e., people with schizophrenia) populations, as shown by several studies (Klinger et al., 2006; Josman et al., 2008; Werner et al., 2009). Josman and colleagues have demonstrated that VAP-S correctly categorized more than 70% of the participants according to their group and initial diagnosis (post-stroke, Minimal Cognitive Impaired and schizophrenics). Moreover, several studies have shown the feasibility of VAP-S as a VR-based tool able to discriminate between controls and these pathological groups, with patients that obtained lower performance in different EFs, such as planning, problem-solving, rule compliance (Werner et al., 2009; Josman et al., 2014). Interestingly, all studies supported a promising convergent validity of the tool due to the correlation between BADS profile score and VAP-S outcome measures (such as trajectory duration, covered distance and time of stops).

#### Virtual Supermarket Shopping Task

Plechata et al. developed the Virtual Supermarket Shopping Task (VSST), a novel solution for assessing and rehabilitating memory and EFs. VSST consists of a simulation of shopping activity set in a small supermarket (29 × 50 m) in which products (e.g., fruits, vegetables) are placed as in a real store. All task was developed using Unity3D software (Plechata et al., 2017). In the VE, the items are visually recognisable, and their names show up to avoid any confusion (e.g., shampoo vs. deodorant). VSST was administered on a 17” laptop, and participants performed the task using a mouse and keyboard. After the exploration phase (maximum of 240 s), where participants could also familiarize themselves with the system, they had to perform two consequent phases: acquisition and recall. During the Acquisition phase, the encoding material (grocery or ordinary supermarket items) was presented to the subject in a shopping list for a specific time (5 seconds for each item). Then, participants performed a delay interval (3 min) without the shopping list and the possibility of moving in VE. After 3 min, the subjects performed the Testing phase, in which they had to find and pick up the stored objects in the virtual supermarket. Participants were instructed to solve the task as fast (short trial time) and as effectively as possible (low trial distance). Interestingly, the examiners could tailor the session to suit the participant's needs, increasing difficulty level (with 3, 5, 7, 9, and 11 items as encoding material). Outcome measures involved the number of correctly collected items and trial time and distance. The errors measured could be composed of two types of mistakes: Intrusion (picking up a wrong object) and Omission (missing some of the objects from the list). Finally, the authors have created two shopping list variants (A and B) for each difficulty level to allow repeated assessment in clinical practice (Plechata et al., 2017). Recently, the authors have conducted a validation study, showing the construct validity of the VSST as a memory task, while further studies are necessary to deepen its validity as an executive function task (trial times and travelled distances – moderate correlations with TMT) (Plechata et al., 2021).

#### VMall

The virtual environment VMall was developed by Rand et al. in 2005 to propose a suitable setting for the rehabilitation of stroke patients in which they had to perform a shopping task (Rand et al., 2005). The authors evaluated the usability of VMall by involving post-stroke individuals showing that VMall has great potential for rehabilitation with patients as it provides an interesting, challenging and motivating task without side effects. Moreover, they affirmed the will to use it again and the great potential for rehabilitation. Interestingly, several patients bought items not on the list because they needed them at home or were on sale; thus, the task appeared relevant and realistic for participants who felt a high level of presence (Rand et al., 2005). The advantage of VR therapy set in VMall was also demonstrated by Jacoby and colleagues in TBI patients, compared to conventional occupational therapy, in improving complex everyday activities (Jacoby et al., 2013). All participants received ten treatments of 45-min, 3/4 times per week. All therapy interventions followed the cognitive retraining treatment that treats and improves deficits in executive functioning through 1) planning tasks; 2) task performance (to perform a task according to planning); 3) time management; 4) monitoring performance; 5) meta-cognitive strategies. In the experimental group, all tasks were performed in the virtual supermarket, and task complexity was adapted to the needs, abilities and progress of each participant. The results showed that 10 on 12 participants improved their performance after therapy. The findings suggested that the improvement in executive functioning was higher in the experimental group than in the control group. Moreover, the study showed that the participants were able to transfer rehabilitation results from the VR treatment to function in the real world, both in similar activities (shopping in the supermarket) and in the performance of additional IADL activities (e.g., cooking). It is possible because the VR shopping simulation tasks were more similar to daily activities than those used during conventional therapy. Finally, the authors supported the idea that the differences between groups may be related to the patients' enjoyment during the intervention that influenced levels of motivation and compliance during the rehabilitation process (Jacoby et al., 2013).

#### NeuroVR Supermarket

Carelli et al. proposed a VR-based tool to treat attention shifting and action planning through tasks that mirrored daily life tasks set in a virtual supermarket developed using NeuroVR software (Carelli et al., 2008). Healthy control subjects underwent a 75-min assessment and training session in which they had to explore the VE, collect some items of a shopping list and listen to any audio announcements that would change the sequence or number of items collected. This treatment involved a hierarchical series of tasks: from a single task condition (level 1) to multiple successive tasks. Outcome measures involved execution times, errors, planning route (trajectories and efficacy) and level of complexity to identify the maximum one that healthy people were able to carry out according to their age range. Results showed the feasibility of the virtual supermarket and attention-shifting paradigm for use with older control subjects. The initial results indicated that the temporal and accuracy outcome measures allowed monitoring differences in these subjects' abilities. Specifically, the execution times appeared to be related to the ability to interact with a computer device like the joypad (as expected). Moreover, the trajectories and efficacy of planning the route can be considered relevant outcome measures. The hierarchical series of tasks allowed clinicians to determine the extent to which adding a contextual and functional executive task interferes with the performance of a simple virtual shopping task by individuals with cognitive impairment. However, the results demonstrate a need to give more practice to ensure that the participants learnt the initial simple task. These promising results paved the way for a subsequent randomised clinical trial and rehabilitative program that addresses additional components of executive functioning (Carelli et al., 2008).

### Virtual Office Environment

Jansari et al. implemented a new instrument, Jansari Agnew Akesson Murphy task - JAAM (Jansari et al., 2004), that uses office environments as scenarios (Jansari et al., 2014). The authors decided to use this context because most of their patients attempted work placement in the office environment. In subsequent studies, the authors named this VR assessment tool with a different acronym “JEF”: Jansari Assessment of Executive Functions; however, the instrument has remained unchanged between the various studies (Jansari et al., 2013).

#### Jansari Assessment of Executive Functions

JAAM reproduces the MET, set in an office environment, to assess eight aspects of executive functioning: planning, prioritisation, selective-thinking, creative-thinking, adaptive-thinking, action-based prospective memory (PM), event-based PM and time-based PM (Jansari et al., 2004). The authors introduced another executive aspect in the JEF version: multitasking (Jansari et al., 2014). The choice of office environment allowed creating a complex task in which participants must complete several tasks in parallel; thus, subjects have to plan and organize their actions to achieve the goals. In this way, the overall assessment is less linear and, therefore, less likely to mask or mediate the difficulties that participants may experience in the workplace. The environment consists of a small office-like room linked by a corridor to a larger room appropriate for holding a meeting for 20 people, that reproduce office and corridor at the University of East London. In these rooms, the authors inserted the items needed for the tasks, additional relevant but non-used items (i.e., staplers and extra desks) (Jansari et al., 2014) and different sounds necessary to replicate the fire alarm and memo announcements (Soar et al., 2016). In the task, participants must play the role of an office assistant with the primary goal of organizing a meeting later that day and preparing an appropriate room for that meeting. The subjects receive a list of tasks that need to be completed for the office manager, called the “Manager's Tasks for Completion,” such as setting up tables and chairs or turning on the coffee machine when the first person arrives for the meeting. They are also informed that they will receive many memos (virtual and hard copy) that require them to perform additional tasks or amend a current task during the task. The responsibility of planning for overall task completion is given to the participant with no clues as to possible solutions or courses of action. The task is presented in a desktop VR environment on a laptop, and the participants can navigate around the environment using the arrow keys on a standard computer keypad and collect objects by clicking them with the computer mouse.

To ensure that performance on the new assessment was not affected by lack of experience with using computers and moving around a VE, participants performed a familiarization phase in a similar VE. After training, each participant is taken to the small office and informed of the role that they must play to complete the task (Jansari et al., 2014). As said previously, the JEF evaluates nine aspects of executive functioning using realistic subtasks (two for each construct) that could be found in an average office environment. The authors designed all tasks with ambiguous and multiple solutions, as in real-life situations (Jansari et al., 2014; Denmark et al., 2019). For clarity, a definition has been inserted for each construct, and some task examples will be described. In the planning scale, subjects must logically order events/objects and not due to their perceived importance. For the prioritization scale, subjects must order events following the perceived importance. In the Selective-thinking scale, subjects must choose between more alternatives by drawing on acquired knowledge. For example: decide which mail company should send each post item based on each company's specialty. In the creative-thinking scale, the user must look for solutions to problems using unobvious and unspecified methods. For example: find a way to cover graffiti written on a whiteboard in indelible ink. For the adaptive-thinking scale, participants must achieve goals again in the face of changing conditions. For example, the overhead projector needed in the meeting is broken and needs to be replaced. In the multitasking scale, subjects must perform more tasks simultaneously. Finally, to evaluate the three constructs of prospective memory, the subjects must remember to execute a task in three different conditions: at a specific future time point (TPM), stimulated by an external stimulus/event (EPM) or stimulated by a stimulus related to an action the individual is already engaged in (APM). For example: turn on the overhead projector 10 min before the scheduled start of the meeting (TPM), note down the times of fire alarms tested before the meeting starts (EPM), make a note of any equipment that breaks or malfunctions during the day (APM).

In total, JAAM/JEF participants have ~40 min to complete the list of tasks in time for the beginning of the meeting. The start and meeting times are written, and participants have a digital clock to monitor the time (Denmark et al., 2019). The only aspects of the test that required physical interaction outside the VE involved filling out specific lists (e.g., the initial to-do list). The examiner observes the entire assessment and completes the evaluation sheet, using a standardized sheet, while the participants perform the activity (Denmark et al., 2019). All subtasks of each construct are scored on a 3-point scale (0, 1, 2), reflecting the participant's efficiency in completing a task. The scores for subtasks of each construct are then summed, and a total percentage score is calculated for each construct. Moreover, a full performance percentage score is calculated for the JAAM by adding raw scores for each construct, dividing the overall possible rating and multiplying by 100 (Montgomery et al., 2010, 2011). The JAAM appeared a promising solution to evaluate executive impairments in subjects with ecstasy-polydrug users (Montgomery et al., 2010) and acute alcohol intoxication (Montgomery et al., 2011). Subsequently, JEF appeared a good and valid ecological tool to evaluate executive dysfunctions in acquired brain injury (ABI) and other conditions (e.g., mood disorders) (Jansari et al., 2013, 2014; Denmark et al., 2019). For example, JEF was able to detect deficits in EFs (e.g., planning and adaptive thinking) in patients with ABI, despite BADS performance being normal. Moreover, the traditional neuropsychological tests (e.g., Digit span, TMT) showed no differences between groups, except for TMT-A. Overall, these promising results confirmed the potential clinical utility of the JEF with frontal lesions, emphasizing the need for ecological assessment in detecting EFs impairments. Recently, Hørlyck and colleagues successfully investigated JEF's validity as an innovative VR-based test for evaluating daily life executive function impairments in patients with mood disorders (Hørlyck et al., 2021). Patients showed impairments in executive functioning compared to the control group in performing JEF. Moreover, JEF scores predicted performance on neuropsychological tests (e.g., TMT, Fluency tests, letter-number sequencing, digit span), indicating that it could be used as an index of EFs.

Due the promising results, Jansari and colleagues developed a parallels version on JEF, addressed to children Jansari assessment of Executive Functions for Children (JEF-C) (Jansari et al., 2012; Gilboa et al., 2019).

#### Jansari Assessment of Executive Functions for Children

JEF-C involves a birthday party and is designed to assess children between 8 and 18 years of age. In this task, the examiner tells the participants that it is their birthday and they must organize their party. The party takes place in a virtual home with three rooms: kitchen, living room and DVD/games room. In this VE, a front door, which participants can open and a back garden with a gate leading to the neighbor's yard are introduced. The participant can move freely around the three rooms, hallway and garden using the computer mouse, and they must perform all required tasks within these areas. Like the adult JEF, there are eight constructs in JEF-C, each of which has an operational definition. For each of these constructs, the authors created realistic tasks that could happen at a child's birthday party to evaluate them as ecologically as possible. For example: in PL, the subjects must rearrange the list of tasks that must be carried out in 3 phases of the party (preparation, development, end); in PR, they must arrange five cleaning tasks for the end of the party. In ST, they must choose which food gives to guests based on their preferences or allergies; in CT, they must find a way to cover the spider drawn with permanent ink on a blackboard (because a guest is afraid of spiders), and in AT, they must find an alternative seating when one chair breaks. The authors designed the tasks with the same characteristics as the adult version: they have ambiguous and multiple solutions. Although most of the tasks are completed in the VE using a standard laptop, for simplicity, some tasks (i.e., selection and planning tasks) are executed in the 'real world' on hard copy. Before starting the evaluation, subjects must move around the house and collect 13 objects for practicing with the environment. Then, they received by examiner an instruction sheet and a biographical sheet of the guests (e.g., food preferences and allergies). Moreover, the participant receives a letter from the parents indicating what they must do: at the end of the reading, the examiner asks the subject to create his “Activity List” card in paper format. The evaluation's real start begins with the beginning of the VR program, as soon as the participant finishes reading the parents' letter. The assessment takes between 30 and 35 min to complete. However, the participant decides when their birthday party finishes; thus, some participants can take longer. Like the adult version, all tasks are assigned on a 3-point scale for success (0-2). To assess inter-rater reliability, two raters simultaneously and independently scored the performance of nine healthy children while performing JEF-C. Data showed very high inter-rater reliability with correlation coefficients between *r* = 0.96 (*p* < 0.001) and 1.0 (*p* < 0.001) for the eight constructs separately and for the overall average JEF-C score (*r* = 0.999, *p* < 0.001) (Jansari et al., 2014). In 2019, Gilboa and colleagues tested the feasibility and validity of JEF-C, as innovative ecologically valid assessment tool for children and adolescents (aged 10–18 years) with ABI (Gilboa et al., 2019). JEF-C showed the presence of severe executive dysfunction in most patients with ABI. Specifically, patients performed significantly worse on most of the JEF-C subscales and total scores, with 41.4% patients classified as having severe executive dysfunction (Gilboa et al., 2019). Recently, the same authors developed an adapted Hebrew version, JEF-C (H) and assessed reliability and validity in the Israeli context, involving typically developing Israeli children and adolescents (aged 11–18 years) (Orkin Simon et al., 2020). Overall, results showed the potential clinical utility of JEF-C (H) as a VR-based tool for an ecologically valid evaluation of executive functioning in Israeli children and adolescents. Expressly, data indicated that JEF-C (H) showed interesting psychometric properties (e.g., acceptable internal consistency) for measuring EFs performance of young Israeli sample (Orkin Simon et al., 2020).

#### Assessim Office

Another VR office task, known as Assessim Office (AO), was implemented by Krch et al. to evaluate several performances on realistic tasks of selective and divided attention, complex problem solving, working memory and prospective memory (Krch et al., 2013). Participants are seated at least 50 cm away from the computer screen and are immersed in the virtual office environment (rendered in the Unity game engine), in which they must navigate and complete virtual tasks using both keys of the mouse. The combination of many tasks of different priorities (e.g., rule-based decision task, reaction time task) is designed to simulate scenarios similar to the real world. In the virtual office, the participants are seated at the virtual desk equipped with several office objects, such as a computer monitor, a keyboard and a file folder. Moreover, the VE includes other desks, two printers, many everyday office objects (e.g., ring binders, lamps, computers, drawer units), a conference room with a projector screen and two big windows. In this task, the subjects must complete several working tasks during a typical workday that lasts ~15 min. Before beginning the task, the examiners show participants the location of crucial objects and what tasks they will perform during their workday. Then, the subject can familiarise with the environment and, as necessary, receive the task instructions again. Participants must carry out five working tasks: 1) respond to emails, 2) decide whether to accept or reject real estate offers based on specific criteria, 3) print the real estate offers that met specific criteria, 4) retrieve printed offers from the printer and deliver them to a file box located on participants' desk and 5) ensure that the conference room projector light remained on at all times. Each task reflects specific EF skills and processes, respectively selective attention, complex problem solving with working memory component (2nd and 3rd tasks), prospective memory and divided attention. In addition to evaluating the task behaviors, off-task behaviors are assessed for the presence of inattentiveness and perseverative behaviors. To facilitate the administration of AO tasks, the authors created an instruction manual that includes many questions frequently asked by participants, with standardized responses and hints for common confusions (e.g., if the participant is lost in the virtual office, the initial cue is “Are you looking for something?”) (Krch et al., 2013). The validation study showed that AO could be an exciting solution for evaluating executive impairments in patients with MS and TBI compared to healthy controls (Krch et al., 2013). The findings suggested a significant difference between patients with MS and the control group on all executive tasks of AO, except for the printing decision task (working memory). Moreover, AO successfully distinguished TBI subjects from controls on specific aspects of EFs: selective and divided attention, problem-solving, and prospective memory. Finally, results showed a good convergent validity due to the relationship between performance on AO tasks and standardized neuropsychological tests (subtests WAIS-III: Letter Number Sequencing and Digit Span; Delis-Kaplan Executive Function System (D-KEFS). Interestingly, a qualitative feasibility assessment revealed that patients could tolerate involvement in a VE with only minimal difficulty moving around the VE with the mouse.

### Virtual Kitchen Environments

Other researchers have investigated the potential of virtual kitchens to assess and train patients with the dysexecutive syndrome (Cao et al., 2009; Klinger et al., 2009; Júlio et al., 2016; Chicchi Giglioli et al., 2019). The use of virtual kitchens can be related to two premises: 1) cooking is a good example of a real-world task that is often based heavily on executive functioning (Tanguay et al., 2014); 2) many assessments and rehabilitation studies of clinical populations have successfully used kitchen settings to address functional and executive impairments (Baum and Edwards, 1993; Zhang et al., 2003; Craik and Bialystok, 2006; Giovannetti et al., 2008; Allain et al., 2014; Ruse et al., 2014).

#### VR-Cooking Task

VR-cooking task is a protocol that uses a virtual kitchen, developed using Unity software as a VE for the ecological assessment of EFs (Chicchi Giglioli et al., 2019). Subjects performed the virtual cooking activity wearing a head-mounted device (HTC VIVE1) and two manual controllers to move hands inside the environment.

Before starting the cooking activity, participants had to familiarize with the technologies, performing an action similar to the virtual cooking activity, to learn the main body movements and hand interactions useful to complete the training. The task begins when they press the button “start.” The virtual cooking task consists of four levels of difficulty that involve three different abilities: attention, planning, and shifting. Each level's main aim is to cook a series of foods at a predetermined time without 1) burning (i.e., food is not removed from the pan or the subject switches off the burner after the predefined cooking time) or 2) cooling (food remains in the pan after it was cooked and turned off the switch, or food is removed from the pan during cooking. Before each level, the users see the instructions about what activities they must perform, how much time each food requires, and the reminder to cook foods without burning or cooling them. When the food is cooked, the participant must remove it from the pan, turn off the cooker, and place it on the plate. Participants could move to the following level as soon as they have cooked all the foods of the previous level. The following levels require more to be completed. In the first level, subjects have to cook three foods in one cooker in 2 min; in the second level, they have to cook five foods on two cookers in 3 min; in the third level, they should perform a dual-task: (a) 5 foods should be cooked on two cookers in 4 min; (b) during the cooking, users should add the right ingredients to the foods, In the last level, another dual-task has been proposed: (a) participants should cook five foods in 2 cookers in 5 min and (b) they should set the table. The virtual system collects the time used to complete the activity at every level, along with total times, burning times and cooling times.

Chicchi Giglioli and co-workers conducted a preliminastudy that evaluated healthy subjects' performance at this ecological task, showing great usability, feasibility, and sense of presence (Chicchi Giglioli et al., 2019). Recently, the same authors have presented a preliminary study to test this task as an alternative to the traditional, standardised neuropsychological tests (e.g., Dot-probe task, Go/No-go test, Stroop test, TMT and ToL) for assessing EFs impairments in patients affected by Alcohol Use Disorder (AUD) (Chicchi Giglioli et al., 2021). Patients with AUD showed lower functioning, with more errors and higher latency times than healthy controls. Moreover, a moderate-to-high relationship appeared between standardized neuropsychological tests and the VCT. Finally, higher relationships were found in the AUD group than the control subjects in the questionnaire evaluating attention control, impulsiveness, and cognitive flexibility, mainly related to planning and cognitive shifting abilities (Chicchi Giglioli et al., 2021). Overall, this study provides initial evidence that a more ecologically valid assessment can be a useful tool to detect cognitive impairments in patients affected by AUD.

#### Therapeutic Virtual Kitchen

Klinger et al. designed the Therapeutic Virtual Kitchen (TVK) as an assessment and rehabilitation instrument for patients with brain injury (Klinger et al., 2009). TVK allows the therapist to adapt virtual kitchen tasks (ecological tasks) to patient abilities, modulating the difficulty (Cao et al., 2010). The authors designed the TVK in collaboration with Kerpape Rehabilitation Center; for this reason, the TVK is graphically very similar to the Kerpape Center kitchen and is based on the habits and needs of Kerpape therapists. Moreover, the authors conducted a preliminary study (2009) involving graduate students or laboratory staff members and therapists who worked with brain injury patients (Cao et al., 2009, 2010; Klinger et al., 2009). This study allowed assessing various conditions of experimentation within the TVK and understanding how to improve the system. Results showed the necessity to add new components such as a final virtual evaluation scale based on the traditional scale used in Kerpape Rehabilitation Center. The virtual activity is displayed on the screen of any computer, and participants can interact, using the mouse, with several 3D objects required in the preparation of meals and can navigate through the environment using the keyboard. To increase the sensation of immersion in the virtual kitchen, the authors introduced real sounds activated with the interaction with 3D objects. Visual mouse signals are given to the subject to facilitate the understanding of interaction opportunities, such as changing the mouse cursor when an item is “pickable” or according to user action (execute, pour, activate, connect). In this VE, participants are involved in virtual IADL (vIADL), such as preparing a coffee. The tasks were designed to meet some fundamental issues related to the primary tasks, the graduation of the task, and the modalities of interaction (Klinger et al., 2009). The TVK software offers two types of activities: “primary” task (PT) and “complex” task (CT). PT has been designed to ensure the participant's familiarisation with the system and tools and to administer simple tasks that can be proposed before involving the patient in the CT (i.e., Coffee task). To perform a PT, the participants must complete a limited number of actions. An example of PT is “take a glass and put it on the table.” CT involves vIADL, namely tasks which require both planning and space-time organisation. Specifically, the CT consists of the preparation of a coffee. The TVK offers the therapist many possibilities to identify the task, adapt it to the participant's skills, achieve the therapeutic goals (evaluation or rehabilitation), and modulate the difficulty. Each task can be modulated by manipulating the 1) the number of cups to prepare (from one to six); 2) time constraint (time organization and stress induction), and 3) initial location of the required items (easy: all the items are ready in the right place; medium: everyone is on the table; hard: need to retrieve all items in the closets or drawers). Due to the various items' locations, the authors worked on the action “take and put”. To transport an object from one place to another, they proposed different solutions: 1) use of an inventory, like in video games; 2) use of “Drag and drop,” like on PC desktop; and 3) stick of the item on the mouse cursor after its selection. The number of steps depends on item location and the nature of the coffee (easy: 12 steps; medium: 14 steps; and difficult: 16 steps). Moreover, based on the activity and the patient's ability, the therapist can help the participant through visual or auditory signals by pressing the keyboard keys (F1: a voice, F2: a message on the screen, F3: a red arrow to indicate the object with which to interact). The TVK can record, in a virtual assessment grid, ten errors: 6 actions errors (AE) and four behavior errors (BE). The AE consists of actions omissions, actions not completed, perseveration, sequence errors, actions additions and control errors. These errors are automatically interpreted and recorded by the system in the virtual assessment grid. The BEs are error recognition, difficulty in decision making, dependence (i.e., patient needs instructions to recall), use of the therapist's help. Before recording these errors in a virtual grid, the therapist must interpret them and press a keyboard key (Cao et al., 2009; Klinger et al., 2009).

In the following year, the authors explored the feasibility of TVK with healthy subjects and seven patients with brain injury (Cao et al., 2010). All control group participants succeeded in completing primary and complex tasks. As regards patients, six patients succeeded in completing the PT and five in completing the CT. Results showed: 1) parameter setting of the configuration (time constraint, number of cups of coffee to prepare, positions of objects) is handy for the therapists; 2) all tasks are understandable and exciting for two groups; 3) virtual interaction is moderately challenging for people who had not computer games experience; 4) helps are comprehensible for patients without expertise. This study showed an issue in recording participants' errors that appeared different between virtual and real assessment grid (e.g., 61 vs. 40) due to different interpretation (e.g., after the patient's correction, therapists interpret still errors whereas TVK does not record “actions omissions”) (Cao et al., 2010).

#### EcoKitchen

Júlio et al. conducted research to get a comprehensive picture of the real-life executive deficits shown by Huntington's disease (HD) patients using a novel VR task – “EcoKitchen” (Júlio et al., 2016, 2019). It was designed and developed to evaluate planning, multi-tasking, set-shifting, cognitive flexibility, self-monitoring, sequencing, divided attention and scanning skills. EcoKitchen is a non-immersive VR task (implemented on a desktop PC) in which participants must perform a VR task (preparing meals) with an increasing executive load that simulates daily-life routines usually done in a kitchen setting. The non-immersive VR task is more portable, facilitating evaluation in clinical contexts (Allain et al., 2014) and limits the risk of cybersickness (Attree et al., 1996; Kawano et al., 2012). The participant experiences a virtual place from a first-hand perspective and uses the mouse to move around the environment. In this VE, the authors introduced a cooking task divided into three blocks with the increasing executive load. A Global Practice Block and training for each block were implemented to ensure that each subject was familiarized with the apparatus before beginning the assessment blocks. In the Global Practice Block, the participant has to explore the kitchen environment and take a specific list of necessary items and distractors. In the first block, the participant has to prepare a cup of coffee with milk (task A). At the bottom of the screen, a list of images with the elements needed to prepare a cup of coffee with milk is shown. The participant must collect each item in the order in which they were displayed in the list as quickly and accurately as possible. Moreover, they have to turn off the stove when the clock is entirely red. To resolve this block, participants must plan and monitor their behaviors.

In the second block, during the execution of task A, the participant must pay attention and control a boiling kettle on the stove. They must press the kettle every time, and as soon as smoke came out and a red signal appeared on the right upper part of the screen to prevent water from spilling. The kettle is programmed to explode three times during the block at random moments. To complete this block successfully, participants must plan and monitor their behavior and divided attention. In the last block, the subject performs the two previous tasks (Task A and boiling kettle) while preparing toasts with butter (Task B). At the bottom of the screen, participants can see a list of images with all the elements needed to make the second snack. Participants are instructed to alternate between the two lists (Task A and Task B) to complete both tasks simultaneously. In this block, the participants must apply the same skills used in the previous block, plus switch/alternate between tasks. To reduce memory workload, the participants can see on full display the instructions and the lists with the items and actions needed to perform Task A or Task B during the whole blocks.

Moreover, to increase task ecological validity, known commercial brands are used to represent the foods and drinks included in the kitchen setting.

EcoKitchen output measures combine time (performance time and reaction time) and error variables, overcoming existing methods based on only one dimension (Giovannetti et al., 2008). In both tasks, the performance time lasts from the first item of the list is picked until the last element of the list is chosen and used. This parameter evaluates psychomotor and processing speed, planning, motor time (Task A and B) and task switching (only Task B). Regarding the reaction time, the authors defined four different parameters: time reaction stove or toaster (to react and switch off the stove/toaster when the clock is entirely red), reaction kettle (to react and turn off the kettle when the smoke appeared and a red signal flashed), time reaction per block (mean of reaction time for each block). The time reaction stove/toaster indicate behavior monitoring, response initiation, divided attention, and set movement. Moreover, the reaction kettle reflects divided attention, prolonged alertness, response initiation, and set changes. Finally, the time reaction for block reflects all the executive subdomains involved in each task. Moreover, the authors evaluate the errors made by each subject: sequencing, item and impulsivity errors). Firstly, the sequencing errors consists of the number of times that the participant failed to follow the proper sequence of the task (i.e., he mixes the coffee with the spoon before adding the milk) and reflects planning, behavior monitoring, and working memory. The item errors represent the number of times the participant collects unnecessary items to prepare Task A or Task B (i.e., he selects a pineapple instead of coffee), measuring attention and behavior monitoring. Finally, impulsivity errors represent the number of times the participant has attempted to turn off the stove/toaster before the correct time (before the clock is entirely red). This parameter reflects response inhibition or inhibitory control, attention and task switching. Total score (total errors/performance time) includes the number of errors per minute that the participants do during the completion of the task and indicates the speed-accuracy balance in the tasks (Task A and B) and switching abilities (only task B).

In a first preliminary study, the authors showed that this VR-based task appeared sensitive to early deficits in both premanifest HD and manifest HD than controls, with clinical groups that showed an overall slower cognitive and motor performance and a higher number of attention errors (Júlio et al., 2016). In the following study, the same three groups underwent complete evaluation with EcoKitchen and traditional neuropsychological batter (e.g., Phonemic Verbal Fluency test, Semantic Verbal Fluency test, Stroop test, Symbol Digit Modalities Test, Digit Span Test, TMT and WCST) (Júlio et al., 2019). Manifest HD group showed deficits in all assessment measures with a statistically significant correlation between the EcoKitchen, traditional neuropsychological battery and HD clinical features. On the contrary, in the premanifest HD group, the executive impairments were only found in the EcoKitchen task (multitasking, divided attention and set-shifting, working memory, planning, monitoring). Therefore, the EcoKitchen task can be considered a sensitive ecological assessment tool for executive functioning in HD, capable of identifying dysfunction symptoms before onset. Interestingly, identifying and quantifying subtle disease-related alterations in individuals who carry the abnormal gene but do not yet meet the criteria for a clinical diagnosis of HD provides a new opportunity for interventions to prevent or delay the onset of symptoms (Weir et al., 2011).

#### Kitchen and Cooking

The game “Kitchen and cooking” is a Serious Games (SG) developed in the context of the European FP7 project VERVE (Manera et al., 2015). Within the topic “virtual reality and executive functions,” SG are digital applications that can be considered a promising non-pharmacological tool to evaluate and treat patients' functional impairments (Robert et al., 2014). Kitchen and cooking is born from the collaboration between clinicians and game designers. In this game, a cooktop is used to evaluate and treat EFs (e.g., planning, attention and recognition of objects) and praxis. The game is installed on a tablet to be used at home and in nursing homes. In this game, participants play different scenarios/recipes. Kitchen and cooking is based on a cooking plot, in which subjects can play four different scenarios/recipes: pizza, yoghurt cake, chicken breast in cream sauce and salmon wrap. In each scenario, participants must: 1) select the right ingredients from the refrigerator and cupboards; 2) plan what actions the subjects must perform and in what order; 3) perform specific gestures for each action (i.e. to rotate finger to mix the ingredients). The first activity supposes research that involves object recognition and sustained attention. The second activity consists of a task that requires planning abilities, while the third activity includes a task that supposes praxis skills. Depending on the scenario, the number of objects to be recognized varies from 5 to 7, the number of activities of planning changes from 5 to 8 and the number of practices ranges from 7 to 13. As the outcome measures, the game keeps track of the time spent in a scenario and the time spent in each game activity. Moreover, it records the total number of scenes played (completed successfully or not) and the number of errors made. The preliminary study confirmed the overall acceptability of Kitchen and cooking, showing that participants (MCI and AD) described the game experience as enjoyable and appeared highly satisfied and motivated by the game, experiencing several positive emotions and not being fatigued (Manera et al., 2015). Overall, “Kitchen and cooking” can be considered a promising instrument to evaluate and rehabilitate executive impairments in elderly people with MCI and AD.

### Virtual City Environments

Other authors paid attention to environments that reproduced entire **cities** or typical public places, such as **apartments** or **libraries** (Jovanovski et al., 2012a).

#### Virtual Library Environment

Renison et al. developed the Virtual Library Task (VLT) (Renison et al., 2008, 2012), a non-immersive VR role-play task that can run on any modern computer using an X-box and PlayStation compatible handset. The VE accurately reproduces the dimensions and contents of two rooms in the Library of Epworth Hospital. In this task, participants must perform several specific activities associated with the daily management of the library, following particular rules. Participants need to decide some priorities and complete multiple activities while managing interruptions, along with novel information that require a change in their behavioral approach. Before beginning the real task, each user is trained in the navigation of the VE for 3–15 min, depending on the participant's experience with VR software. The VLT comprises functional tasks designed to reflect and evaluate seven components of executive functioning that reflect different theoretical models and factor analyses about EFs (Damasio, 1996; Busch et al., 2005; Crawford and Henry, 2005; Stuss, 2007; Testa et al., 2012). Specifically:

1) Task Analysis through a) Identify the most logical and efficient order to perform the tasks on the “To-Do List.”; b) Identify the most efficient order for the library books to be delivered to members' homes while ensuring the task rules have adhered. 2) Strategy generation and regulation through a) generate an alternative solution to cooling the library when the subject is informed that the air conditioner doesn't function; b) identify how to photocopy a 3-page document when only two pieces of paper are available; c) generate an appropriate solution to place 8 cups on the table when only 7 cups are placed on the tray; d) create a suitable solution to connect three devices when only two power points are available. 3) Prospective Working Memory by a) selecting the most appropriate catering menu in light of a series of required criteria; b) selecting the five most relevant elements for a library display given a set of specific criteria needed. 4) Interference and dual-task management through a) perform another task while waiting for the photocopier to warm up; b) stop a less critical task when an urgent job is presented and, after that, return to the first task. 5) Response inhibition by a) following the task rule by inhibiting the automatic response of answering the telephone; 2) respecting the task rule of not making personal phone calls when phone messages say to do it. 6) Time-based Prospective memory through a) turning the computer on at exactly 8:55 am; b) note the no arrival of the food at 8:57 am. 7) Event-based Prospective memory through four functional tasks: a) remove books from the sheet of loan when books are returned; b) place book on table when it is returned; c) document the time in which a specific book is returned; d) control the tray when passing by the library desk.

Participants should complete the tasks in 20 min: impulsivity and the abilities of planning and problem-solving will influence the time taken to resolve the tasks. The examiner used operationalized scoring criteria (three-point criteria 0, 1, 2) to rate the functional tasks accurately completed. The functional tasks mapped on the seven components of EF, weighted proportionately according to the number of functional tasks that mapped onto them. Outcome measures include seven subtask scores ranging between 0 and 8; thus, the Total Score ranges from 0 to 56: low scores reflected reduced EF.

To obtain data about the intra- and inter-rater reliability of the VLT, the performance of 11 participants was videotaped and rated by two independent raters both at the time of task administration and 1 week later. Preliminary data showed strong inter-rater reliability (rVLT = 1.0; *p* 0.001) and strong intra-rater reliability (rVLT = 1.0; *p* 0.001) (Renison et al., 2008). In their study, Renison et al. (2008) compared the Virtual Library VLT, the Real Library Task (RLT) and neuropsychological measures of executive functioning (e.g., Verbal Fluency, WCST), involving patients with TBI and healthy subjects. Data showed a correlation between VLT and the RLT, indicating that VR performance is similar to real-world performance. Moreover, VLT could discriminate between two groups, with patients that obtain less performance in prospective memory and interference tasks. On the contrary, most of the neuropsychological measures failed to differentiate the groups (Renison et al., 2008). Therefore, VLT can be considered a good ecological measure of EFs in TBI because it showed a superior ability to differentiate between patients and controls, even after controlling for age, education, intelligence, and verbal memory.

#### Multitasking in the City Test

Multitasking in the City Test (MCT) is another VR executive task developed by Jovanovski et al. to evaluate planning and multitasking abilities ecologically (Jovanovski et al., 2012a).

MCT consists of an errand-running task that takes place in a virtual city that involves participant's home, offices (post office, doctor's office, optometrist), bank, restaurant, dry cleaners, coffee shop and many different stores (stationery store, pet store, drug store, grocery store). The number of places inside the city is complex enough to represent a typical commercial centre but not too difficult to confuse the task from a spatial perspective. This VE was displayed on a standard personal computer without a head-mounted display (HDM). It was viewed from a first-person perspective, without a graphical representation of the subject in the environment. Participants can navigate through a standard joystick and make action decisions by pressing number keys on the keyboard. Subjects can freely enter all buildings, but interactions are only possible for those buildings that are part of the activity requirements. The arrangement of the shops in the virtual city is not accidental: shops in which the participants had to make purchases are close to the subjects' house and far to the bank, where they had to take the necessary funds to complete those purchases. At the bottom of the monitor, participants can view a list of the errands they must perform, the errands completed, the amount of money in their pocket, the items in their backpack and a clock that displays the time (in hours, minutes, and seconds). All tasks in the MCT were developed to be similar to the types of tasks most people engage within regularly (i.e., shopping, attending appointments, etc.). Participants initially underwent training of 3 min in which they must familiarize themselves with the joystick and navigate around the city. During training, they may pay attention to where stores and other buildings are placed in the town. After that, participants receive instructions about the tasks to complete: “It is now 4:30 p.m., and you are at home. You are planning to spend the next 15 min or so running errands within your city of residence. The following errands are on your list of things to do: 1) You must go to various stores to purchase the following items: six blue pens, which cost various prices; cough syrup, which will cost $4.00 and groceries, which will cost a total of $12.50. All of these items can be picked up directly at the cashier: the pens are normally found there, and the other items have been pre-ordered and have therefore been placed at the cashier's area for you to pick up. 2) You must go to the doctor's office as you have an appointment for 4:40 p.m. You may arrive early, but you cannot be late; 3) you must pick up your mail from the post office; 4) at the post office, you have to drop off a letter for which you must buy postage, which will cost 75 cents. 5) You must go to the bank to withdraw money as you only have 67 cents in your wallet. You must try not to go over your budget of $20.00 for all the items you need to buy today; 6) you should be back at home no later than 4:45 p.m.” In summary, the examiners required participants to purchase several items, obtain money from the bank and attend a doctor's appointment within 15 min. These various test elements introduce complexity in the test situation: successful performance depends on judicial and common-sense decision-making rather than a more simplistic approach. Before starting the evaluation, the examiner can repeat the instructions and answer possible questions. Next, participants must construct a plan of how they will complete the task using the map: they are provided with a pen and paper to record their program. Participants are provided with unlimited time for planning because it is interesting to explore how planning time may be associated with the task performance variables (i.e., completion time, number of tasks completed, errors made). The plan's quality will help assess organizational abilities and evaluate if plan quality is correlated with task performance. In the MCT, the subjects must perform tasks with few explicit rules, unlike tests such as the VMET, in which participants must follow specific rules (Raspelli et al., 2009). The authors suggested that including rules could mask or reduce monitoring and other executive deficits by providing more structure within the test situation, as traditional tests. So, the only rule specified in the MCT is that the users must return to their virtual home within 15 min to avoid excessive testing time. During this time, participants must perform and complete as many of the eight commissions as possible: this number was considered a realistic number of tasks that a person would typically perform in everyday life. The tasks in the MCT is relatively simple, with little variability in the level of complexity of the individual tasks, but the order that subjects must choose to perform the task may represent a challenge. To perform a task, the subjects must explore the VE using a joystick; when they arrive near to place, a screen will prompt them to choose what they would like to do from a list of options (i.e., “buy an item,” “withdraw money,” or “Nothing”). Each option corresponds to a specific number, so participants will have to press the appropriate number from the number keys on the keyboard. When a task is completed, it will appear under the “Tasks Completed” section of the screen, and all of the items they have acquired will appear in the “Items in Backpack” section of the screen. Moreover, to help subjects navigate around the city, a map of the town is beside them at all times (Jovanovski et al., 2012a).

The following outcome measures can be obtained from these tasks: Completion Time (in seconds), number of Tasks Completed (maximum score of 8) and Task Repetitions. Moreover, each participant's test is recorded, and a video file of task performance is used to determine qualitative errors. The qualitative errors can be categorized as Task Failures, Task Repetitions, or Inefficiencies. The Task Failures involve 1) insufficient funds errors that consist of the number of times in which participants tried to make a purchase for which they had insufficient money in their pocket, 2) incomplete task; 3) purchasing an expensive item over the more economical option (i.e., $4 pens VS $2 pens); 4) not meeting the scheduled time deadlines (i.e., not attending the doctor's appointment on time or not returning home on time). The Inefficiencies consists in 1) entering a “task” building but not carrying out the activity; 2) performing the post office task in two separate visits instead of one visit; 3) entering a building but not performing a task; 4) notable wandering behaviour (walking around the town at least 30 s without executing a task and not necessarily involving visits to any buildings). Finally, the Task Repetitions consist of achieving the same task more than once and on two separate occasions: 1) attending doctor's appointments two times; 2) acquiring the same item more than once; 3) withdrawing money from the bank more than once. Errors are scored separately by the same two independent raters: the intraclass correlation coefficients computed for each failure category are all perfect (1.00), indicating excellent interrater reliability. Moreover, each participant's plan on how to complete the activity is assessed with a score between 1 and 6, basing on a logical sequence.

After developing MCT, Jovanovski and colleagues showed the ability of the test to investigate planning and multitasking in healthy subjects, comparing it with traditional neuropsychologic tests for executive functioning (e.g., TMT, ToL, BADS–Modified Six Elements Test, Wechsler Test of Adult Reading, Judgment of Line Orientation) (Jovanovski et al., 2012b). The preliminary results suggested that the MCT might provide an ecologically valid method of objectively evaluating EFs since data showed a significant correlation between MCT scores and traditional paper and pencil tests (convergent validity). Moreover, the authors showed the efficacy of the test in discriminating executive functionality between patients with Acquired Brain Injury (ABI) and healthy controls, particularly patients who showed more problems in initiation (Jovanovski et al., 2012b). The initiation problems could be responsible for the “knowing and doing” dissociation (Alderman et al., 2003): a good plan did not necessarily translate into successful task performance (patients complete fewer tasks than the control group). In other words, patients showed good comprehension of the task requirements (“knowing”) as evidenced by their planning, but they did not start the tasks indicated within the planning (not “doing” tasks). Overall, MCT may offer a superior method of evaluating the degree and nature of real-life executive function impairments compared with traditional EFs measures.

Recently, Newman and co-workers have conducted a study using a VR cityscape to assess how immersion within an environment (developed using Unity) that one appraises to be dangerous impacts concurrent executive function performance (Newman et al., 2020). Participants were immersed in this VE using a VR-HDM and a mouse to answer tasks. Within the VR cityscape, participants were virtually placed in a chair either at ground height (Low VR height) or atop a pole several stories above ground (High VR height). The task asked subjects to perform two executive function tasks (response inhibition and updating) in low and high height scenarios. In the response inhibition task, participants completed an auditory version of a Go/NoGo task [four 3-min blocks, with 200 trials per block]. They had to respond as fast as possible to the “Go” stimulus (a 550-ms 900 Hz sine tone) and hold the answer to the ‘NoGo' stimulus (a higher pitch sine tone - 550-ms 1,200 Hz). In Updating task, participants performed an auditory 2-Back task to engage working memory updating processes [each block lasted about 3 min, 125 trials]. Stimuli, spoken letters (A-E), were presented in a random order for 1,000-ms, followed by silence for 1,000-ms. Participants responded with a mouse-click when a letter was the same as that heard previously (“2-back”). Each trial lasted 2,000-ms, and responses were counted if they occurred within 50–1,800-ms of the beginning of the stimulus presentation. Preliminary results revealed that EFs were impaired when participants were placed at a virtual high height, but only for those with self-reported negative appraisals of heights. This study demonstrated that VR could be used to understand real-time performance in life-like situations, providing a foundation for building strategies that can be used to protect cognitive performance during threatening or anxiety-provoking situations (Newman et al., 2020).

#### Virtual Apartment Environment

##### Virtual Apartment Environment - Virtual Reality Day-Out Task

In 2013, Tarnanas et al. designed the “Virtual Reality Day-Out Task” (VR-DOT), a fire evacuation task developed to improve the ecological validity of EFs assessment, using a verisimilitude approach (Tarnanas et al., 2013) VR-DOT permits to detect marked impairments in cognitive performance (particularly in EFs) doing activities that resemble ADL (VR-ADL). The VR-DOT consists of a fire evacuation drill exercise composed of 6 different simulated fire situations of increasing difficulty (from easy to more difficult). The aim is to investigate the performance, in an experimentally controlled manner, on a complex ADL (planning and evacuating a fire under time pressure) that is more indicative of the real quality of seniors' life. This task takes place in a virtual apartment block with three floors and five apartments per level. Participants must prioritize, organize, initiate, and complete several subtasks to evacuate safely from an apartment level (second floor) to the ground area. For example, they must collect and select information on the size of the fire and avoid smoke. The VR-DOT examines prospective memory and reasoning in a complicated emergency routine. To design, create and develop this complex task, the authors used many hardware (e.g., Pentium-based computer), software (e.g., Maya software), sensors (e.g., LEAP motion sensor) and tools (Kinect camera).

Moreover, the authors used the Microsoft Kinect software development kit (Microsoft Corp, Seattle, WA, USA) to analyze gestures and movements and develop a user interface system. User tracking was performed by a “Flexible action and articulated skeleton toolkit” (FAAST; University of Southern California, CA, USA), a middleware to facilitate the integration of full-body control with games and VR applications. In their study, Tarnanas and co-workers provided preliminary evidence of ecological and construct validity of this VR-based instrument as a screening tool of physical and cognitive abilities in early dementia (Tarnanas et al., 2013). VR-DOT allowed investigating in a controlled environment the performance in complex ADLs (planning and evacuating a fire under time pressure) that indicate seniors' real quality of life. The findings showed that the VR-DOT had great sensitivity and specificity as screening tests in discriminating amnestic MCI, mild AD and normal ageing by detecting differences in errors, omissions, and perseverations. Interestingly, this VR-based tool allowed evaluating functional impairments at a very early phase of AD, showing good psychometric properties (i.e., discriminant power) to contribute to a pre-dementia diagnosis (Tarnanas et al., 2013).

##### Virtual Apartment Environment - Edinburgh Virtual Errands Test

Another task also used to evaluate some executive measures in a real-life context, such as planning and intentionality (i.e., goal-directed behavior), is the Edinburgh Virtual Errands Test (EVET) (Chen and Hsieh, 2018). The EVET was developed in 2011 by Logie et al. to reproduce everyday multitasking activities in a VE (Logie et al., 2011). Everyday multitasking involves several subtasks (which involve several cognitive processes) with different requirements and how people program these subtask attempts. For this reason, the EVET includes a range of cognitive functions acting together, such as retrospective and prospective memory, working memory, planning and intentionality. EVET was created using the Software Development Kit (SDK) supplied with the computer game Half-Life 2™. The SDK can be used for free for the non-profit development of VEs, allowing to create realistic environments, design actions according to the researcher's needs, and obtain an automatic recording of multiple performance measures. The environment includes a 3D model of a four-story building with rooms on the left and right of each floor around a central stairwell with two sets of stairs (one left, one right) and a central elevator. Participants must complete eight errand tasks efficiently within 8 min while navigating this simulated environment. They can explore the VE, displayed on a standard computer, using the standard keyboard (keys “w,” “s,” “a,” and “d” for forward, backward, left and right movements, respectively), and the mouse to look in any direction (all participants' movements were recorded at 10 Hz). Moreover, they must use the “e” for actions such as collecting items or opening doors (Logie et al., 2011). Before beginning the task, each participant must read the instructions and specific rules in 2 min. They receive the following instruction: ”Please imagine that you are a student and are assigned to make a list of errands for your teacher. The errands are listed in a particular order, but you can vary the order at any time as you wish. However, you are also told not to enter any of those rooms unless the rooms are on the list. You have 8 min to complete these assignments. Please complete all the assignments as soon as you can, and complete as many as possible.“ Moreover, they receive these three rules: 1) Use left stairs for travelling down and right stairs for travelling up; 2) do not pick up non-task-related objects; 3) do not enter non-task-related rooms. After that, the participants must perform four practice errands in about 5 min: 1) object collection and delivery, 2) button pressing, 3) unlocking the stairwell door with a key code, and 4) folder sorting. These errands allow participants to familiarize themselves with the building. Then, they receive one of the two errand lists with the eight errands (List A or B) to complete in 8 min. For each list, three chores consist of two-stage: object collection and drop off, while the remaining five require one action. Two tasks have time constraints while sorting folders is an open-ended task. In list A, the eight tasks are: 1) Pick up the brown package on the Third floor (T) 4 and take it to the Ground floor (G) 6; 2) Pick up the newspaper in G3 and take it to the Desk in S (second floor) 4; 3) Obtain the keycard in F (first floor) 9 and unlock G6 (*via* G5); 4) Meet the person in S10 before 3:00 min; 5) Obtain the stair-code from the notice board in G8 and unlock the stairwell; 6) Turn on Cinema S7 at 5:30 min; 7) Turn off the Lift G Floor; 8) Sort the red and blue binders in room S2. The list B involves: Pick up the computer in G4 and take it to T7; 2) Pick up mil carton in T3 and take it to the Desk in F4; 3) Get the keycard in S9 and unlock T7 (via T6); 4) Meet the person in F10 before 3:00 min; 5) Get the stair-code from the notice board in T10 and unlock the stairwell; 6) Turn on Cinema F7 at 5:30 min; 7) Turn off the Lift T Floor; 8) Sort the red and blue binders in room F2. Participants were informed that folder sorting is no more important than other tasks but that they should try to sort as many as possible at any time during all test time. In the EVET experiment, participants have 2 min to study their errand list, followed by the free recall (in total approx. 4 min). Then, another 5 min of further study of this errand list and a cued recall test (in whole approx. 7 min). The subjects receive the errands in a non-optimal order for completion, so they must plan, in about 7 min, the order in which they should perform each errand to achieve the task effectively. The eight tasks can be performed by interleaving or switching from one to the other when each task is completed. Next, participants receive a schematic building map and a copy of the errand list, and they must indicate the order in which they planned to perform each errand (subjects are informed that they can change their plan during the test). After that, they must verbally recall the errand list and rules without any suggestions (errors are corrected) until they can remember 100% of the list (at most 15 min). This last procedure is implemented to minimise the possibility that participants fail to complete errands because they don't remind all errands. As soon as they recall 100% of the list, participants must begin the EVET task and perform the errand list (A or B) in 8 min, without a task list or plan. On completion, they must recall the complete errand list: the errands they had attempted or failed to complete and any building rules they had broken. Moreover, they are cued about any errands they had omitted, along with the errands correctly recalled in this post-test recall (in total approx. 5 min). Finally, participants receive the other list of errands, and they must plan the most efficient sequence of errands (planning task) without performing the EVET a second time (approx. 7 min) (Logie et al., 2011). A general EVET score is calculated based on participants' overall performance, accounting for completed errands and incorrect actions. In particular, the EVET score indicates errand completion efficiency: points are added for each errand completion and bonus points are awarded based on the number of folders sorted and the time discrepancy for timed errands. Moreover, points are removed for breaking building rules: picking up incorrect objects, entering rooms not on the errand list and breaking the stair rules. Bonus and penalty points are given on a five-point scale (0-4) based on a cut-off score (Logie et al., 2011). The rating can rate between 12 and 20. Moreover, the EVET subscores are calculated: for example, to measure the efficiency of navigation through the building, the authors calculated the “EVET travel time”: the total time that the participant has spent travelling in the EVET building, excluding time spent inside rooms. Besides, the authors evaluate the pre-test and post-test plans (plan score before and after the EVET test), and the plan follows; if the planning and the actual completion order are the same, the participant receives 1 point. The other measures are classifiable with labels taken from Burgess and colleagues (Burgess et al., 2000): 1) Lean (errand list memory score): the sum of the scores of free and cued recall of errands (before the task). The maximum score for the errands was 42 (1 point for each recall, correct). 2) Recount (remind all actions): after the EVET test, participants are immediately asked to recall errands completed or failed and any violations of the rules. If participants could recall a keyword, they received 1 point. The maximum possible score was 28. 3) Remember (errand list memory score): the sum of the scores of free and cued recalls of errands (after the task) (Logie et al., 2011). In a preliminary study, Chen and Hsieh (2018) investigated if individuals with frequent internet gaming (IG) experience exhibited better or worse multitasking ability compared with those with infrequent IG experience (Chen and Hsieh, 2018). The results showed that subjects in the frequent IG group obtained a better performance on EVET. However, the performance of both groups on the conventional laboratory task (e.g., dual-task and task switching) did not differ significantly. Thus, the frequent IG group showed better multitasking efficacy only when measured using a more ecologically valid task (Chen and Hsieh, 2018).

As regards Virtual Apartments, the other two authors conducted preliminary studies in which participants had to perform several increasingly difficult everyday tasks, set in the small virtual apartment consisting of an entryway, a kitchen, a dining room, a living room, a bathroom, and a bedroom (Baumann, 2005; Banville and Nolin, 2012). Both studies have shown how these VEs could be used for a more ecologically valid study of EFs with head-injured patients. Future studies will have to be conducted to deepen the efficacy of VR-based tools in a clinical population.

### Virtual Classroom

The Virtual Classroom (VC) is an immersive VR system (that requires a Head Mounted Display, HMD), developed and validated by Rizzo et al. to assess attentional skills in children through increasing difficulty tasks and varying levels of distraction in a VE (Rizzo et al., 2000, 2004, 2006; Sharkey, 2004). For example, the Stroop task is one of the many attentional and executive tasks that can be easily performed by projecting stimuli into the VC blackboard and via the teacher's voice (Rizzo et al., 2000). Overall, the tasks have to involve items that measure attention in a sophisticated manner without requiring complex reading, language and reasoning skills. In the VC task, participants sit at a virtual desk in a virtual rectangular school classroom including desks, a blackboard on the front wall, a teacher, other students and a large window looking out onto a playground and street with moving vehicles (Rizzo et al., 2006; Gilboa et al., 2011, 2015). Moreover, the environment was settled with classroom distractors: classroom noises and movement of virtual classmates or cars in the street (Parsons and Carlew, 2016). Thus, participants must perform the cognitive task with auditory, visual and audio-visual distractors in the background. The attention ability can be measured through performance on a variety of attention challenges that the examiner can modify based on the age or education of participants. For example, the children must press a “colored” section of the virtual desk upon the teacher's direct instruction or when she pronounced a specific colour (focused or selective attention task). Moreover, the examiner can manipulate the test time to evaluate sustained attention. Finally, the examiner can use a more complex task to assess alternating or divided attention: the child must press the “colored” section only when the teacher pronounces the color about an animal and only when the word “dog” is written on the blackboard. VC environments present auditory or visual distractors in various areas of the simulated classroom. The auditory distractors involve the sound of an aeroplane passing overhead, a voice from the intercom, the bell ringing, paper crumpling, a pencil hitting the floor, a sneeze and a cough. The visual ones involve paper planes flying in the room. Finally, the audio-visual distractors include a school bus or car driving by the book dropping to the floor, children passing notes, a child raising his hand, the teacher answering the classroom door and the principal entering the room (Parsons, 2015). The examiner can adjust these distractors' number, frequency, and characteristics (i.e., sounds of vehicles) according to age, education, or other testing needs (Parsons and Carlew, 2016). Moreover, the examiners can modify some aspects of the VE, such as the seating position of the subject, the number of virtual students and the sex of the teacher (Rizzo et al., 2006). This complex system can run on a standard processor, such as the Pentium 3 processor with the NVIDIA G2 graphics card (Sharkey, 2004). For clarity, two examples of tasks assigned in a VC will be described.

A) The first task was developed by Rizzo et al. (2000). In this VE, three conditions were presented for9 min each. In the first one, the subject had to press the response button, only when the letter appeared on the blackboard was “X” preceded by an “A.” The examiner administered the same task in the second condition, adding some distractions. The stimuli used in the first two conditions were not typical of a classroom environment. In the last condition, the cognitive challenges involved attention tasks typically found in a classroom environment with visual and auditory sensory stimuli. In this condition, the subject had to follow verbal instructions from the virtual teacher that directed attention to the blackboard, where visual stimuli requiring a response appeared. For example, the virtual teacher requested to press the button if an image of a cat appeared on the blackboard. So, in this condition, a variety of “real-life” classroom stimuli and tasks can be created using auditory (teacher's speech) and visual (on the blackboard) presentation of colours, geometric forms, numbers, letters, single words, complete sentences, and illustrations of objects.

In 2006, Rizzo et al. successfully showed the possibility to integrate the VR classroom in assessing children with Attention-Deficit/Hyperactivity Disorder (ADHD) (Rizzo et al., 2006). Specifically, the VR-based tool showed that children with ADHD had slower correct hit reaction times on the distraction condition and higher reaction time variability on correct hits on both conditions than healthy controls. Moreover, children with ADHD made more omission errors (missed targets) and commission errors (impulsive responding in the absence of a target) on both conditions. Finally, the exploratory analysis of motor movement in children with ADHD (measured with eye-tracking technology) indicated higher activity levels on all metrics than non-diagnosed children across both conditions (above all in distraction condition): children with ADHD missed targets due to looking away from the blackboard during 25% of the trials (vs. 1%).

B) Gilboa et al. in two studies designed another task: in these versions of VC, the authors used digits instead of letters, and the test instructions are respectively in Hebrew and French (Gilboa et al., 2011, 2015). The task required participants to view a series of numbers on the blackboard and press the mouse button as quickly and accurately as possible, using their dominant hand, when the digit sequence “3 followed by 7” appeared on the blackboard. They were required to hold responses to any other series of digits for 10 min. The stimuli remained on the screen for 150 ms, with a fixed inter-stimulus interval of 1,350 ms. The test lasted for 10 min, with five identical blocks of 2 min each. Specifically, 400 stimuli were presented, of which 100 were “3-7” sequences. These stimuli were accompanied by 20 distractors, displayed for 5 s each. These distractors were auditory (i.e., pencils dropping), visual (i.e., a paper aeroplane flying across the visual field) and mixed audio-visual (i.e., car “rumbling” by a window). Outcome measures depend by the task used, but generally, they involve different aspects of attention: correct and incorrect response (response variability – sustained attention), commission errors (incorrect identification of the non-target as the target - impulsivity), reaction time and head-turning (hyperactivity) (Sharkey, 2004; Gilboa et al., 2011). The authors successfully proposed the VC to evaluate attention processes in a Neurofibromatosis type 1 (NF1) population (Gilboa et al., 2011), showing that patients are more inattentive (omission errors) and impulsive (commission errors) than healthy controls. On the contrary, no significant differences were noted between groups on the reaction time to targets and head movements; thus, the attention deficits of children with NF1 do not include more overall hyperactivity. In the following years, Gilboa et al. used the same VC task to evaluate children with ABI compared to controls (Gilboa et al., 2015). Results showed that the VC appeared a sensitive, playful and ecologically valid assessment tool for diagnosing attention deficits (above all sustained attention) in children with ABI (Gilboa et al., 2015).

### Virtual Version of Traditional Paper-and-Pencil Tests

As previously said, EFs are traditionally assessed with theory-based neuropsychological tests, which guarantee standardised scores (Stroop, 1935; Nelson, 1976; Reitan, 1992b). However, several studies have shown how these tests appear to be unable to reliably predict the “complexity” of executive functioning in real-life settings (Shallice and Burgess, 1991; Goldstein, 1996; Chaytor and Schmitter-Edgecombe, 2003). Over the years, several authors have developed and implemented virtual versions of the traditional paper-and-pencil instruments (Parsons et al., 2011, 2013; Armstrong et al., 2013).

#### Tower of London

Campbell et al. (2009) implemented the virtual version of the traditional test: Tower of London (ToL) (Shallice, 1982). The authors developed the virtual three-dimensional ToL task to measure brain activity during the performance on a traditional test of planning (Campbell et al., 2009), using Discreet's three-dimensional modelling software, 3DMax. As the examiner could also administer this instrument in Magnetic Resonance Imaging (MRI), an HDM MR-compatible were added to present the visual scene to the subject in the magnet. To follow the original version of this task, the examines required subjects to place the balls on a grid of three pegs (of varying height) according to some goal indicated on a stimulus card. The subject had to move only one ball at a time to a correct place (where space is permitted) and achieve the goal in the least number of moves possible. To execute the task, the subject has to use the LUMItouch device that consists of two paddles (one for each hand) with two buttons on each paddle: these four buttons are used as selecting devices. The first three buttons (from the left side) select the top-most ball in the three respective positions of the ToL pegs. When the subject picked a ball, it became illuminated to indicate it had been chosen. After that, the participants must tap the same first three buttons to indicate the destination of that ball. If the selected destination is invalid (i.e., it is impossible to insert another ball on a particular peg), the item is deselected. On the contrary, if the chosen location is correct, the subjects see the ball's movement to that location. In some cases, the examiner can modify the original ToL task lightly to work on the planning component of the subject's performance. The examiner can suggest participants plan all sequences of moves for 15–20 s before moving the first ball or spend the same time looking at the display without planning anything. These two opposite conditions of planning and non-planning permit isolating planning performance (subtraction procedures). In their study, Campbell et al. also compared the VR version of the ToL with a new ecological task of planning (Campbell et al., 2009). The novel ecological task involved a VR city in which a roadblock task was developed. The VR city was designed within a three-dimensional simulation platform called WorldUp. This city included a variety of buildings: offices, commercial and residential structures, numerous blocks and corridors. In particular, the authors introduced virtual participant home, doctors' offices near the medical centre, workplace, a factory, a hotel, government offices next to legal office and banking institution, a daycare and a supermarket next to the retirement centre. To increase the realistic nature of the VE, the authors decided to include many realistic elements using 3DMax software or Adobe Photoshop (e.g., sky, roadblocks, sidewalks, roads, streetlights, parking lots, cars, park space and trees). In both the learning and execution phase, participants must navigate the VR city through a joystick interface while watching their first-person movements on a monitor (Campbell et al., 2009).

Before starting the three learning sessions, the examiner presented to the participant a hypothetical scenario on which the task was based: the subject had obtained a new job in a new location, and he had to familiarise himself with his new city. During the training sessions, participants must learn two specific paths that they will have to travel to get to the new job: 1) from his virtual home to the daycare and then to his workplace; 2) from his workplace to a supermarket (buying milk or bread) and then to his virtual home. In the first learning session, a computer program automatically shows the participant exactly the first path. If participants make a errors, the examiner has to stop them and re-demonstrates the path through the computer. When the subjects were able to navigate the first path accurately three times, they then had to learn the second path in the same way. Subsequently, when the subjects could also navigate the second path, they must explore the remainder of the city (familiarity task) to know the environment and streets. To make the exploration easier, the participant can access a map by pressing the “m” key on a keyboard (similar to the paper map in the real world). This last phase self-terminates when the subject visited enough of the city. This familiarisation is necessary so that the individual has some implicit knowledge of the entire city to use during the roadblock-navigation of the testing phase. All these learning sequences (except the initial visualisation of the path) are also performed in the two subsequent training sessions. The hypothesis is that the subject can remember the way from his previous training session. During the testing phase (that can be performed in MRI), the examiner initially says to the participants that they have to perform the same spatial navigation task accurately. Then, the examiner gives a novel set of instructions, in which he says that during the navigation, the subject may encounter a construction roadblock, which will obstruct his path. The authors placed strategically eight roadblocks throughout the city, four for each path. They were located strategically to elicit the highest effort when planning an alternative path. The roadblock appears as an arrangement of construction pylons with a pocket in the centre. The participant must approach the roadblock and enter the pocket: it becomes immobile, and one of two construction signs appears, permanent roadblock (2) or temporary roadblock (2). For the temporary roadblocks, the participants see the sign “Temporary delay, please wait,” while for the permanent roadblock, the sign is “Road closed, plan an alternative route.” With a permanent roadblock, the subject must spend the next 15–20 s planning the most efficient alternative route, which can only involve travelling on roads or sidewalks. After these seconds, the roadblock sign disappears, and he can move freely to re-navigate according to the route planned during the delay. If the roadblock is temporary, the participant has to rest and not think about anything in particular; after 15–20 s, the roadblock completely disappears, and he can continue along his original route. The difference in neuro activation patterns between these two states is integral to planning performance. In a preliminary study, one healthy subject underwent three training sessions and an fMRI scanner testing session. Results showed that the subject displayed a great sense of presence while interacting within virtual environments. The image data suggested both convergent and divergent specificity between the two conditions (Roadblock task vs. V-ToL) in location and brain activation intensity. However, the V-ToL task elicited a more widespread and generally higher brain activity level; thus, the VR ToL task appeared more multifactorial, confirming that the ToL is a specific measure of planning and an index of frontal lobe function or dysfunction. Further studies will have to conduct to evaluate the efficacy of this VR-based tool in discriminating between healthy and clinical populations (Campbell et al., 2009).

#### Trail Making Test: Virtual Reality Color Trails Test

Plotnik et al. developed and assessed the validity of a full-body 360-degree VR version of the “Trail Making Test - TMT” (Plotnik et al., 2017). The TMT is a classical EF test of selective attention/task switching used in research and clinical assessments (Reitan, 1992b). Specifically, for simplicity reasons, the authors used a valid variant of the TMT, the Color Trails Test (CTT) (D'Elia et al., 1996). The VR Color Trails Test (VR CTT) consists of two significant parts: subjects are required firstly to 1) connect, in ascending order, circles containing numbers (Trail A); secondly to 2) execute the same task but alternating between two colours (Trail B - for example connecting in order 1 - yellow - 1 rose - 2 yellow - 2 rose…). In particular, VR CTT involves four subparts: practice A, test A, practice B and test B. In this virtual version, the 2D page of the original CTT is replaced with a 3D VR space that introduces a new depth dimension to the target balls with numbers (that correspond to circles with the numbers of the original version) and to the generated trajectory by the subject. The ball size varies based on its proximity to the participant in the VR space in the VR environment. Moreover, the first and last balls are indicated through a hand icon. The participant's performance is documented not via a pen or pencil but through a marker affixed to the tip of a short pointing stick held by the participant. The marker's movements are real-time tracked by a motion capture system at a sampling rate of 120 Hz, which allows the reconstruction of kinematic data over the all-test duration. A virtual representation of this fixed pointing stick marker appears in the visual scene as a small red ball. Real-time visual feedback of the movement in the VR space is generated by a thick red trail, while the movement of the participant's hand tracing the CTT targets is visualised in real-time through a subtle yellow path. Also, four makers are affixed to a headband placed on the participant's head. These markers provide head rotation and translation information within a room-based coordinate system. The primary outcome measures involved Trail A time, Trail B time and the difference between Trail B time and Trail A time that is considered a valid measure of selective attention sensitive to cognitive impairment. In their preliminary study, Plotnick and colleagues provided initial data on the construct validity of a 360-degree VR version of the classic TMT, involving healthy volunteers that completed both pen-and-paper and VR versions of the CTT (Plotnik et al., 2017). Results showed correlations for VR CTT and traditional CTT for Part A, B and B-A, suggesting a good convergent construct validity of the novel VR CTT (Plotnik et al., 2017).

Recently Plotnik et al. developed other two VR-based versions of the Color-Trails Test (CTT): one for a large-scale VR system (DOMECTT) and one for a portable HDM VR system (HMD-CTT) (Plotnik et al., 2021), exploring the effects on motor and cognitive function on 147 healthy adults. Results indicate average correlations among the pencil-and-paper CTT and the VR adaptations of the task. Moreover, VR versions demonstrated significantly high test-retest reliability and discriminant validity. In conclusion, the study of Plotnik et al. shows the feasibility and validity of converting a neuropsychological test from pencil-and-paper to a three-dimensional VR-based format for studying cognitive-motor interactions enhancing the ecological validity of the neuropsychological assessment (Plotnik et al., 2021). Further studies will have to be conducted to evaluate VR-based tools' efficacy in discriminating between healthy and clinical populations.

#### Wisconsin Card Sorting Test: Look for a Match

Elkind et al. developed the Look for a Match (LFAM), a three-dimensional, stereographic scenario aimed at evaluating executive control processes, in which participants performed a task similar to Wisconsin Card Sorting Test (WCST) set in a virtual beach scene (Elkind et al., 2001). Participants are asked to deliver frisbees, sodas, popsicles, and beach balls to bathers sitting under umbrellas. Each umbrella has one of the four objects on it, differing in type, colour, and number (i.e., one beach ball or two frisbees or three soda or four popsicles). While delivering the objects, the subjects receive verbal feedback (i.e., “That's it” or “That's not what I want”); they should use this verbal feedback to identify the rule that will have to guide the next delivery. The matching pattern follows the one of the WCST: participants must match 10 times to color, 10 to object, and 10 times to number to complete the task successfully. The simulation ends when the participant successfully finishes two complete series or after 128 turns. The scene can be presented as a stereographic, three-dimensional computerized beach scene or a two-dimensional version of the same scene. In the virtual version, participants must wear lightweight stereographic eyewear with or without regular eyeglasses. Outcome measures involve total correct responses, total and percentage errors, and absolute and percentage perseverative responses (behaviors or verbalizations driven by unconscious or neurological causes, continue well-beyond interpersonal or circumstantial appropriateness). Additional measures are perseverative and non-perseverative errors (total and percentage), conceptual responses (total and percentage), categories completed, failure to maintain set and learning to learn. In a pilot study, 63 healthy subjects performed both tests, starting alternately with WCST or LFAM. Results showed that the participants found LFAM more enjoyable and exciting but WCST easier. A significant correlation appeared in the execution scores of the two tools, except for perseverative errors. Thus, the LFAM seemed to evaluate the same aspects of EFs of the WCST. Overall, results indicated that LFAM can be a helpful measure of the EFs and might offer a more ecologically valid assessment of executive functioning than the WCST since it reflects real-world situations (Elkind et al., 2001). Other works will have to be conducted to evaluate this VR-based tool's efficacy in discriminating between healthy controls and the clinical population.

#### Stroop Test

The measurement of supervisory attentional control is typically evaluated by the Stroop Test that places task-relevant information in conflict with task-irrelevant information (Stroop Color Naming Task- (Stroop, 1935). Several paper-and-pencil versions of the Stroop task have been designed and developed to evaluate executive functioning and inhibitory control through the presentation of blocks of multiple Stroop stimuli on a card (Delis–Kaplan Executive Function System (D-KEFS) color-word Interference Test (Delis, 1997) or through a single-item presentation of Stroop stimuli (Davidson et al., 2003). Moreover, many authors developed computer automated assessments of single item Stroop, such as Automated Neuropsychological Assessment Metrics (ANAM) Stroop (Johnson et al., 2008). However, both the multiple paper and pencil versions and the computerized version of the Stroop lack various distractions that may occur in daily activities that may diminish predictions about real-world functioning (Spooner and Pachana, 2006; Chan et al., 2008; Parsons, 2015).

An initial attempt to deal with these ecological validity problems consisted of developing and validating the VR Stroop task (VRST) (Parsons et al., 2011, 2013; Armstrong et al., 2013).

##### Virtual Reality Stroop Task

The VRST is one of the assessments included in the Virtual Reality Cognitive Performance Assessment Test (VRCPAT). Like the pencil-paper version of Stroop, VRST presents Stroop stimuli (single-object presentation) to evaluate simple attention, coarse reading speed, divided attentional skills and executive functioning, allowing the evaluation of the reaction time to single-element presentations as the computerized version ANAM. However, conversely to the ANAM and the paper-and-pencil versions, the VRST was developed to respond to the need for militarily relevant tests (Parsons et al., 2011). For this reason, in the VRST, the authors introduced a simulation environment with military relevant stimuli in high- and low-threat settings developed using Maya software (Parsons et al., 2013). The VRST was designed to measure supervisory attentional processing (executive functioning) in the real-world environment with and without cognitive and affective distractors. In this task, participants are immersed in a highly mobile virtual multipurpose wheeled vehicle (HMMWV - virtual Humvee) using an HMD while Stroop stimuli appear on the windshield of a military Humvee while the Humvee automatically drives down a desert road in Iraq. In this task, the authors used two monitors: one to view the Launcher application, which the examiner uses in the test's administration and another to display the participant's view on the VE in the HMD. Moreover, the authors used a sophisticated program to improve the ecological validity and sense of immersion of VR-based instruments, such as accelerator and brake pedals under the table. The environments were rendered in real-time using Gamebryo 3-D graphics engine and MATLAB scoring program (Wu et al., 2010) and human-computer interface (Clinical Neuropsychology and Simulation Interface; CNS-I) are used for data acquisition, stimulus presentation, psychophysiological monitoring and communication between the psychophysiological recording hardware and the VE (Parsons et al., 2011, 2013; Wu et al., 2013). In VRST, participants ride in a simulated Humvee through alternating low and high threat zones. In low-threat zones, little activity in the VE was presented besides driving a desert road. In the high-risk areas, the authors introduced many stressors (e.g., gunshots, explosions and screams). The participants experienced three low-threat and three high-threat zones to manipulate arousal levels: start section, palm ambush, safe zone, city ambush, safe zone and bridge ambush. The VRST was used to manage cognitive workload levels and was completed during exposure to high and low threat areas. The VRST consisted of 4 conditions: 1) word-reading, 2) colour-naming, 3) interference, and 4) complex interference (Armstrong et al., 2013; Parsons et al., 2013). In the first three conditions, stimuli are continually presented in a fixed central location on the windshield, while in the complex interference condition, the Stroop stimuli are offered randomly throughout the windshield. Each Stroop condition was experienced once in a high threat zone and once in a low threat zone. The VRST requires an individual to press one of three computer keys to identify each of three colours (i.e., red, green, or blue). Stimuli occur for 1.25 s each, and participants must respond as quickly as possible without making mistakes. The presentation speed of individual stimuli depends on the user: the next stimulus doesn't appear until the appropriate key is pressed on the color-coded keypad for the currently viewed stimulus. The VRST is a timed measure with a maximum of 50 stimuli available per zone. Data collected include reaction time, response time and accuracy for each area and condition. The color–word score is calculated by (a) multiplying the number of correct colors named by the number of right words called and (b) dividing the product by the sum of the number of accurate colors named plus the number of exact words named. The simple interference score is calculated by taking the number correct on the Interference task and subtracting the color–word score during the simple presentation of stimuli. The complex interference score is calculated by taking the number correct on the interference task and subtracting the color–word score during the elaborate presentation of stimuli (Parsons et al., 2013).

In 2013, Armstrong and co-workers proposed the VRST as a VR-based assessment tool for the military to accurately detect brain injuries (Armstrong et al., 2013). Results showed significant correlations between VRST and computerized computerized (ANAM) and traditional tests (D-KEFS) of executive functioning (convergent validity). However, the mean response times for the VRST were considerably longer than other tests since tasks performed in a virtual environment may require additional cognitive demands than traditional neuropsychological assessments (thus, longer response times) (Armstrong et al., 2013). Generally, the VR Stroop task appeared a helpful evaluation tool for the military because cognitive tasks in a virtual context may better duplicate natural conditions (and situations) of the military field, providing a realistic assessment of impairments. This VR-based assessment opened new lines of inquiry into the impact of environmental stimuli on performance, offering promise for the future of neuropsychological assessments used with military personnel.

##### The Virtual Apartment Stroop Task

Along with developing and validating studies about VRST, Henry et al. (2011) started creating another VR Stroop Task set in a virtual apartment. This VR-Stroop task was developed in collaboration with Digital MediaWorks to obtain a complete inhibition task and improve the sensitivity of impulsivity evaluation (Henry et al., 2011). In this Virtual Apartment Stroop Task (VAST), Stroop stimuli are shown on a television in a virtual apartment (Henry et al., 2012; Parsons and Barnett, 2018, 2019). The participants are seated in a virtual living room in front of the flat-screen television. Many objects, such as the candles on a table and a sofa, were introduced in this virtual living room. Under the TV, various realistic elements were inserted, including a cabinet with a video recorder and DVDs. On both sides of this cabinet, two musical speakers are also located. Moreover, the authors introduced a kitchen (at the left of the television) and a picture of the outside (through a window at the TV's right). The distracters appear throughout the virtual apartment environment. The subjects can view the VE using an HMD that allows participants to look 360° around themselves and explore the virtual apartment environment by turning their heads. In this task, the examiner must use two monitors: one to view the startup application, which the examiner manages the test and another to see the participant's view on the VE in the HMD. The task builds on the unimodal Stroop (Stroop effect) and measures cognitive interference with go-no-go components (reaction time, commission errors, omission errors, and reaction time variability) (Henry et al., 2011). The VAST extends the traditional Stroop paradigm by including bimodal (auditory and visually mediated) stimuli (external interference). This task involves two different conditions: a block-based condition (Color Naming) and a word-based (Word Reading) condition. In the Color naming condition, a series of coloured rectangles (red, blue and green) appear pseudo-randomly on the television while a female voice mentions the names of the three colours (bimodal presentations). The order of colours presented is consistent for each participant. They must click the left mouse button with their preferred hand as rapidly as possible when the spoken colour (audio stimulus) corresponds to the rectangle's colour on the television (visual stimulation) and retain the answer if the colours do not match. In the word-based condition, colour words appear on the virtual television (red, blue, and green) in different ink colours (red, blue, and green). These stimuli can be congruent (i.e., the word “blue” in blue ink) and incongruent (i.e., the word “blue” in red ink). Also, the colours are pronounced by a female voice in this condition. The participants must click the mouse when the stated word corresponds to the colour of the word presented on the television and hold the answer if the pronounced word and presented ink colour do not match. The word-based condition must measure cognitive interference (Stroop effect) in addition to the external interference control and motor inhibition (go-no-go variables) assessed by the block-based condition. The complete duration of the Virtual Apartment-based Stroop was 9.6 min: each task condition lasts 4.8 min with a 1,000 ms inter-stimulus interval (ISI). In each condition, a total of 144 stimuli are presented, with 72 targets (go responses) and 72 non-targets: the 72 goal consists of 36 congruent and 36 incongruent stimuli. The participants execute both no-distraction (congruent color named and figure/word viewed/read) and distraction (incongruent - interference) conditions for each task's condition (block-based and word-based) (Henry et al., 2012). During the interference condition, distracters (auditory, visual or audio-visual distracters) can appear in different field of view locations in the VE. On the left of the visual field, only auditory distracters (i.e., doorbell, pencil dropped and sneeze) appear. On the contrary, on the right, the subjects can see both auditory (i.e., vacuum cleaner and jackhammer outside of the building) and audio-visual distracters (i.e., school bus and SUV that pass on the street and are viewed through the window). In the centre, subjects can see all three types of distractors: visual (woman walking in the kitchen), auditory (jet noise that passes over the building) and audio-visual (i-phone ringing and vibrating on the table, Toy Robot on the floor and Kit-Cat Clock with wagging pendulum tail and revolving eyes). Moreover, they can see a paper plane flying from the room's left to the right. Data collected include 1) mean and variation of total reaction time (simple and complex - i.e., the latency of response); 2) mean and variation of reaction time for correct answers; 3) correct responses (accuracy) and 4) performance (correct responses per minute of available response time; Thorne, 2006); 5) commission errors; 6) omission errors (Henry et al., 2011).

In 2012, Henry and colleagues developed a Bimodal VR-Stroop set in a Virtual Apartment as an innovative tool for measuring selective attention, control of cognitive interference, and motor inhibition (Henry et al., 2012). This task extends the traditional Stroop paradigm by including two conditions (block-based and word-based) and bimodal (auditory and visually) stimuli. In their study, the authors evaluated several impulsivity/inhibition measures of healthy volunteers with: (1) the conventional Stroop task (D-KEFS), (2) the Elevator Counting task (ECT) with distraction (Robertson et al., 1994); (3) the Continuous Performance Task (CPT) II; (4) the Stop-it task (Verbruggen et al., 2008) and (5) the VR-Stroop task (Henry et al., 2011). Preliminary data showed that the VR Stroop with a bimodal presentation of the stimuli could elicit the Stroop effect. Moreover, Bimodal VR-Stroop scores (included commission errors and reaction time) correlated significantly with impulsivity measures (ECT, CPT and Stop-it task). Furthermore, the bimodal VR-Stroop seems capable of measuring internal interference control and motor inhibition simultaneously via simple reaction times, omissions, and commissions. Interestingly, participants reported few side effects (e.g., eyestrain) and a good sense of presence. Overall, these preliminary results showed that Bimodal VR-Stroop could represent a short (10 min), enjoyable, portable, and multi-component assessment of inhibition, including selective attention, control of internal and external interference (Stroop effect) and motor inhibition (reaction times) (Henry et al., 2012).

In 2018, Parsons and colleagues compared the performance of 91 healthy undergraduates in three different Stroop Tests: (a) the traditional paper-and-pencil Stroop Task - D-KEFS, (multi-item, unimodal); (b) computerised Stroop - ANAM (single-item, unimodal); and (c) the Virtual Apartment-Based Stroop (single-item, bimodal Stroop stimuli in a simulated apartment) (Parsons and Barnett, 2018). Results showed that the performance appeared more poorly on the Virtual Apartment Stroop task when distractors were present. Moreover, Virtual Apartment Stroop allowed evaluating the accuracy and total time of performance, reaction time, and distractors' impact on participant performance simultaneously. Finally, Virtual Apartment Stroop allowed overcoming the limits of paper-and-pencil and computer-based assessment by providing scenarios that reflect everyday activities in controlled environments. In the following year, the authors showed the potential of a Virtual Apartment Stroop Task to distinguish prepotent response inhibition and resistance to distractor inhibition in ageing adults, with older subjects that obtained poorer performance (less accuracy and longer reaction times) (Parsons and Barnett, 2019).

##### The Virtual Classroom Stroop Task

The Virtual Classroom Stroop Task is another virtual task based on the Stroop Test to evaluate EFs. As previously said, VC was first developed by Rizzo et al. (2000). It was revised by the Digital MediaWorks team (http://www.dmw.ca/) under the name ClinicaVR: Classroom-Stroop. In this VR platform, Stroop stimuli appear on a virtual blackboard in front of a VC (Lalonde et al., 2013; Parsons et al., 2013; Parsons, 2015; Parsons and Carlew, 2016). During the task, the participants sit at a virtual desk in a virtual school classroom containing many realistic elements such as desks, a blackboard, a window, a teacher and other students. An HMD is placed on students' heads (eMagin Z800 with an InterSense InteriaCube 2+ attached for tracking), allowing entire head movement with a complete 360° view of the VC. A tracking device connected to the visor allows transferring locational information to a standard computer. This computer enables updating the images presented to the user and increase the illusion of immersion in a real environment. To process the ClinicaVR Classroom program, the examiners can use a standard desktop computer. Before each task, an avatar teacher reads the instructions in front of the virtual class. The participants must repeat the instructions and perform a short practice trial (Lalonde et al., 2013). As in the Virtual Apartment Stroop Task, the task consists of two conditions: colored boxes and colored words. In both conditions, inter-stimulus intervals of 1,000 ms are used. In the first condition, boxes of three different colors appear successively on the virtual blackboard. When the stimulus occurs, the teacher pronounces a color: if the color pronounced corresponds to the blackboard, participants must click on the mouse. In the second condition, color words written in different shades of chalk appear. The stimuli can be congruent (i.e., the word BLUE written in blue chalk) or incongruent (i.e., the word BLUE presented in red chalk) (such as in the third condition of the D-KEFS Color-Word Interference test). The participant must click on the mouse only when the color indicated by the teacher corresponds to the color of the chalk. The VC allows participants to respond physically through a single response, which enables the evaluation of motor inhibition. The bimodal presentation of stimuli in the VC Stroop task would assess the participant's ability to control interference from external (e.g., environment) and internal sources (e.g., judgments). The addition of distractors in the VE would allow the evaluation of external cognitive inhibition by requiring participants to resist distractions in the environment (Lalonde et al., 2013).

The convergent validity of this VR-based tool was evaluated by Lalonde et al. (2013), involving 38 healthy adolescents that completed Stroop Test set in VC and five D-KEFS subtests (Trail Making, Tower, Twenty Questions, Verbal Fluency and Color-Word Interference). The results showed a promising correlation between performance on the VR Stroop task and standard EFs assessment. Furthermore, a significant correlation appeared among the number of rule violations and commission errors on some subtests of D-KEFS (measures of individual's capacity to follow instructions and inhibit inappropriate responses) and performances on both conditions of the VR-Stroop task. Results also showed that VR-Stroop performance more accurately reflected everyday behaviors and executive functioning (evaluated with two questionnaires completed by parents: Behavioral Rating Inventory of Executive Function and the Child Behavior Checklist) than paper-pencil tasks. Generally, VR offers an ecological assessment of everyday functioning and could be linked to standard tests to evaluate cognitive abilities (Lalonde et al., 2013).

##### Virtual Classroom Bimodal Stroop

Parson and colleagues developed and evaluated the Virtual Classroom Bimodal Stroop (VCBS) that measures cognitive interference go/no go components (assessing motor inhibition) and external interference control (accomplished via visual and auditory distractors) (Parsons, 2015). In this version, participants are immersed in the VC and must answer Stroop stimuli while many auditory (school bell; coughing, dropped pencil) and visual (paper-aeroplane flies across the room, school bus passes outside the window, students passing notes) distractors occur in the VE (Parsons, 2015; Parsons and Barnett, 2018). The VCBS task is similar to Lalonde's version and the Virtual Apartment Stroop task. Like the other two versions, it consists of two conditions: a block-based condition and a word-based condition. However, in this task, a female voice pronounced the colours (vs teacher avatar), and the task's non-distraction or distraction condition is introduced. In this version, for each condition, a total of 144 stimuli are presented, with 72 targets and 72 non-targets. The duration of each condition is 4.8 min with a 1,000 ms inter-stimulus interval (as in VAST). Outcome measures involved in both versions of the VC (Bimodal) Stroop task are reaction time, the response time (reaction time for correct responses), omission errors, commission errors (used to evaluate the inhibition capabilities) and accurate answers (Lalonde et al., 2013; Parsons, 2015). In their study, the authors compared a VC Bimodal Stroop task (VCBS) with paper-and-pencil (D-KEFS) and computerized (ANAM) Stroop in individuals with typical development and with Autism Spectrum Disorders (ASD) (Parsons, 2015). Results indicated that the classic Stroop pattern occurred in traditional modalities and the VCBS task. Moreover, data showed individuals with ASD obtained significantly worse performance in the VCBS task with distractors, but they didn't show in other traditional tests and VCBS task without distractors (Parsons, 2015).

Dahdah et al. showed that an immersive VR treatment could improve executive dysfunction in patients with brain injury (Dahdah et al., 2017). In their project, the authors involved both VR-Stroop (ClinicaVR: Apartment Stroop) (Henry et al., 2011) and Bimodal VR-Stroop (VR Classroom) (Parsons et al., 2007). This treatment consisted of 8 VR sessions (2/week for 4 weeks) that differed in the quantity and type of distractors. Participants were immersed in the VEs using a 3D HDM and answered the VR stimuli through a mouse. Session 1 (baseline) included all types of distracters (auditory, visual, audio-visual) simultaneously. Sessions 2 and 3 included no distracting stimuli. In the following sessions, the authors introduced distracters varying by sensory modality to evaluate the increased executive burden. Specifically, they presented only distracting auditory stimuli in sessions 4 and 5 and only distracting visual stimuli in sessions 6 and 7. The last session resembled baseline by including all distracters again to evaluate the change in performance. The first and last sessions lasted ~60 min (vs. 30 min) because they included the assessment of EFs using the traditional neuropsychological tests (e.g., D-KEFS and ANAM). Overall, results showed that patients with brain injury improved in sustained attention, attention to visual details, cognitive flexibility and impulsive errors across sessions that included virtual apartment, while information processing speed improved across sessions that involved the VC (Dahdah et al., 2017). Overall, these promising findings supported the ecological validity of immersive VEs in capturing and treating executive deficits in patients with neurological dysfunction.

#### Continuous Performance Testing

##### Virtual Reality Continuous Performance Testing

In 2018, Areces et al. (2018) developed an innovative VR version of the traditional paper-and-pencil Continuous Performance Testing (CPT) (Rosvold et al., 1956), reproducing the conditions of a regular classroom. This tool assesses children's attention, impulsivity, processing speed, and motor activity. The test activities are predetermined, and the examiner cannot modify any characteristic of the tasks. Before starting the test, the examiner must require participants to wear the HMD and the headphones: the glasses are connected to the PC, so the therapist can see the images the participants are looking at in real-time. Moreover, the participant receives a button necessary to respond during the tasks. During all the phases, the virtual teacher will provide participants with all necessary instructions and guide them through the tasks. Firstly, participants must explore the classroom carefully and take the perspective of one of the students sitting at desks looking at the blackboard for 15 s. The virtual teacher introduces this first part: “Hello, with the glasses that you are wearing, you can see the entire classroom: to the left, to your right, up, and down. You can see everything. Notice all the things in the room, look at the walls and the other people, look at whatever you want.” In the VC, the participant may see many desks, a blackboard on the front wall, a teacher, other students, many specific school objects (e.g., books, notebooks or map) and a large window looking out onto a street with buildings, tree and moving vehicles. After that, the participant has to perform training, which consists of visually locating four red balloons placed around him (by moving his head) and popping them by pressing the button. Then, the subject must perform two exercises, designed according to the original CPT paradigm: vigilance tasks (activation mechanism, known as X - task) and inhibition tasks (inhibition mechanism, known as x-no tasks). In the first one, which is based on the “x-no” paradigm (traditionally known as “no-go”), participants must press the button as fast as possible only when they do not see (on the blackboard) or hear the stimulus “apple.” For example, they must press the button every time they hear “cloud” or see a cloud drawing on the chalkboard. Subsequently, they have to perform another exercise that follows an “X” paradigm (or “go” task). Here, the subjects have to press the button whenever they see (on the blackboard) or hear “seven.” At the end of the test, the examiner obtains a report with these results: omissions, commissions, response time and variability. Moreover, this information is differentiated according to sensory modality (visual vs. auditory), presence/absence of distractors and task type (go vs. no-go), thereby leading to different execution profiles. In their preliminary work, Areces and colleagues showed that VR-based CPT, as a paper-and-pencil version of the test, was able to detect lower scores in attentional variables (i.e., omissions, commissions, response times and motor activity) in subjects with ADHD compared to healthy controls. Moreover, they showed lower cortical activation and blood oxygenation in frontal brain regions during the administration of this VR-based tool in patients. In general, this protocol could provide a more realistic and reliable assessment for the diagnosis of ADHD and offer recommendations for parents and teachers, more adapted to each child's individual needs (Areces et al., 2018).

##### Advanced Virtual Reality Tool for the Assessment of Attention

Advanced Virtual Reality Tool for the Assessment of Attention (AULA) is another VR test designed to assess attention among children and based on the CPT paradigm (Iriarte et al., 2012), also used for diagnosing ADHD. Conversely to standard tests, AULA is presented as a VR “game,” thus facilitating the initial predisposal of children and adolescents to the evaluation. This task is based on Sergeant's state regulation model (Sergeant et al., 1999): state regulation can be considered an EF, affected by the frontal lobe and its connection with the limbic system. AULA needs a particular set of VR glasses with movement sensors and a button to respond to different tasks. The environment represents a primary or a high school classroom: the participants sit in one of the classroom desks and, from their perspective, can see many desks, a chalkboard on the front wall, a teacher, other students and many specific school objects (e.g., books, notebooks or map). As CPT, AULA consists of two main exercises: a No-X task (overstimulation) and an X-exercise (hypoactivation). In the first one, participants must respond to the non-target stimuli and ignore target stimuli, for example, “press the button when you do not see or hear apple,” on the contrary, in the second one, the subjects must answer to target stimuli and ignore non-target stimuli, such as “press the button whenever you do see or hear seven” (Iriarte et al., 2012).

The authors chose this sequence for the exercises because it reproduces the child's self-regulation problem more accurately: the difficulty in adapting to new environmental needs after performing an over-stimulating activity. Stimuli are presented both in a visual and auditory modality. In the first exercise, the stimuli involved are a tree, bottle, book, apple and cake, while in the second one, the targets include some numbers (fine, six, seven, eight, nine). Moreover, the authors introduced ecological visual and auditory distractors, such as the teacher walking through the room, object drop-off, environmental noises. Before administering the test, the participants must be familiar with technology (VR glasses, switches, audio headset, etc.) to minimise anxiety associated with the evaluation context. They receive the technological devices and listen to audio instructions: “Hello, welcome to AULA, with the glasses you are wearing, you can see the whole classroom, at your left, right, up and down… you can see everything.” Then the voice describes the classroom environment and the type of stimuli and tasks presented. Before performing the real exercises, the subjects must execute brief training tasks, with two aims: (a) to familiarize with the type of tasks that will be performed subsequently; (b) to avoid a state of over motivation or anxiety due to the use of this type of novel technologies. The complete administration of AULA lasts ~20 min. After each administration of AULA, the virtual teacher tells the child to remove the VR glasses. AULA allows the analysis of the behavior and information processing abilities in both tasks, with or without distractors. In particular, with this new tool, it is possible to obtain several measures correlated to different aspects of attention: omission errors (child does not press the button when he should - selective and focalized attention); commission errors (child presses the button when he should not have to - impulsivity); reaction time (time to answer a stimulus, not only when the answers are correct) and motor activity (necessary vs. unnecessary movements by the movement sensors placed in the VR headset). Moreover, these variables can be considered as a general measure or categorized by the sensorial modality of the overlooked stimulus (visual vs. auditory), by the influence of distractors (presence vs. absence), by the type of task (No-X task vs. X task). Besides, the information obtained allows for differences between visual and auditory processing skills and between No-X and X tasks (i.e., overstimulation and hypoactivation tasks). In their validation study, Iriarte and colleagues involved a normative sample of 1,272 healthy participants between 6 and 16 years. Results evidenced promising differences according to gender, age and type of stimuli. Firstly, regarding gender, males provided faster answers (both correct and incorrect—commission errors) and performed greater motor activity (more head movement) and greater deviation from the focus. On the contrary, girls appeared slower in providing answers but obtained better performance in all types of tasks and conditions. Secondly, the age differences appeared in the initial age groups, whereas they were not so evident in the following age groups, especially after 10 years old (participants between 12 and 16 years presented stable attentional parameters). This stability may suggest the normal development of cognitive processes measured with this test. In conclusion, visual and auditory attention differences appeared since the 6-year-old group. The visual omissions and commissions were more frequent than auditory ones, and the time required to offer visual answers was shorter both for correct responses and commission errors than the time needed for auditory ones (Iriarte et al., 2012). Further studies will have to evaluate the convergent validity of AULA, comparing it with traditional standardized tests (such as CPT) and the efficacy in discriminating healthy controls and patients with executive dysfunctions.

##### Nesplora Aquarium

Voinescu et al. have also used the CPT to develop a VR system - Nesplora Aquarium- to assess attention and EFs in adults (Voinescu et al., 2019). This instrument involves the vigilance CPTs (AX-types) administered in a virtual aquarium. In contrast with the inhibition CPTs, the vigilance CPTs require participants to answer to target stimuli and ignore non-target stimuli (Rosvold et al., 1956). During the test phase, they see a virtual aquarium, and they have to perform different CPTs composed of visual stimuli (e.g., various species of fish that are passing through two rocks, placed in the main fish tank) and auditory stimuli (e.g., names of the different species of fish). The participant must follow a specific rule for answering stimuli, but this rule changes between the various tasks. For example, in the first task, the users have to press a Bluetooth-paired button every time they see or hear a specific fish name (e.g., clownfish), but only if another type of fish was caught or its name was heard before (e.g., surgeonfish). The authors adapted Baddeley's dual-task paradigm (X) to the CPT framework for the other two tasks: participants must respond differently to targets based on visual and auditory stimuli. To increase complexity, many different distractors (auditory and visual) were introduced separately or simultaneously. The visual distractors involve people walking in front of the aquarium and other animals present in the aquarium (e.g., turtles), while the auditory ones include an invitation to coffee delivered over the PA system, a baby crying, a warning to not use the flash when taking photos. Four task versions were developed during the field trials to achieve acceptable difficulty and reliability levels. In the final version, the stimulus interval is 500 ms for the visual stimuli and 770 ms for auditory stimuli. Inter-stimuli interval is pseudo-randomized between 1,500 and 2,000 ms. So, in total, the participant spends about 20–25 min in the VR aquarium. This VR aquarium is displayed through a VR headset that uses a Samsung Galaxy S7 smartphone, paired with Samsung Gear VR goggles and headphones. The examiner can monitor the test through a laptop computer connected to VR using a local wireless connection (Voinescu et al., 2019). Outcome measures involve omission and commission errors, reaction time variability, and motor activity. After the VR exposure, the subjects completed the System Usability Scale (Brooke, 1996) to measure self-reported usability and learnability. Healthy participants rated Nesplora Aquarium as good to excellent (a grade and a percentile rank of 90–95). Hence, the VR system can be considered more usable than 90-95% of products (Sauro and Lewis, 2016). This is a promising result that highlights the potential of this VR-based tool for neuropsychological evaluation (Voinescu et al., 2019).

In 2020, Camacho-Condea and Climent successfully tested the effectiveness of this VR-based tool in assessing attention and working memory involving adolescents (60 with ADHD diagnosis and 60 healthy controls) (Camacho-Conde and Climent, 2020). Specifically, significant differences appeared between two groups in processing speed, selective attention, and cognitive inhibition: general execution, attention arousal and processing speed. Recently, Voinescu et al. tested the effectiveness of Nesplora Aquarium in assessing attention and inhibition in participants with low and elevated symptoms of depression and anxiety (Voinescu et al., 2021). All participants performed the Virtual continuous performance test in which they had to respond to stimuli (fish) in a virtual aquarium and traditional tests for executive functioning (e.g., Continuous Performance Test, Stroop Test, Corsi Test, TMT) and symptoms of depression (Beck Depression Inventory-II - Beck et al., 1996) and anxiety (State-trait Anxiety Inventory - Spielberger 1983). Results showed that participants' performance in VE was positively associated with classic measures of attention and inhibition, allowing clinicians to evaluate symptoms of depression and anxiety not detected by traditional measures, such as psychomotor speed and spatial working memory. Moreover, the VR-based tool distinguished between participants with elevated and low symptoms, with the first displaying overall poorer attention performance (i.e., reduced vigilance, increased inattention and psychomotor slowness). Finally, the authors evaluated the system usability, sickness and sense of presence in VE, showing good promising results for both groups (Voinescu et al., 2021). In conclusion, the Nesplora Aquarium can be considered a secure, usable and ecologically valid assessment tool, able to detect deficits in attention, working memory and inhibition in different clinical populations (Camacho-Conde and Climent, 2020; Voinescu et al., 2021).

Overall, this paragraph showed that the VR-based instrument showed a good convergent validity compared to corresponding traditional paper and pencil tests. However, few studies have evaluated the usability of these tools and their efficacy in discriminating between control subjects and clinical conditions.

#### Virtual Executive NEuropsychological REhabilitation Project

In 2002, Lo Priore et al. developed the Virtual Executive NEuropsychological REhabilitation (V.E.Ne.Re.) Project. The project aimed to plan, develop and test a rehabilitative protocol for EFs through the construction and validation of artificial environments based on VR technologies (Lo Priore et al., 2002). Their study started from the difference between performance in non-immersive traditional tests and real-life of frontal patients. The VEs became a promising alternative to enhance the neuropsychological assessment and rehabilitation of executive functioning since they offer evaluations and treatments in environments that reproduce real-life situations. The project provided several steps in which the authors had to plan and realize the VEs according to the real needs of patients, analyzing their usability, engagement and sense of presence with patients and healthy controls and, finally, their efficacy in the rehabilitation. Within this project, the authors proposed an innovative VR-based tool for rehabilitation, “V-Store,” which consists of several tasks to empower EFs, programming, attention, short term memory, behaviour control and metacognition (Lo Priore et al., 2003). Moreover, the project involved a VR version of Shallice's ToL (V-ToL) and a VR version of the WCST (V-WCST) (Lo Priore et al., 2002; Castelnuovo et al., 2003) that used the same VE of V-Store, but the original paradigms and trial sequences are carefully respected. Both tasks can be used one-time as assessing tests or repeated as a rehabilitative instrument (the examiner can intervene on all variables implied). As their original versions, the V-ToL evaluates the executive ability to program behaviour in time (Shallice, 1982), while the V-WCST turns to the executive skills of categorisation, abstraction and behavioural flexibility (Heaton, 1981). The VE consists of an internal fruit store where participants perform increasingly complex tasks. The subject views his avatar (his representation in the virtual world) in front of a conveyor belt on which some baskets (from one to three) cross the room. The participants must explore the VE and fill up the baskets with pieces of fruit that they can find on four shelves placed on the room's walls. In the beginning, the subject receives a verbal command through a loudspeaker situated on the front wall, which instructs him about what to do: how to fill the baskets and with what kind of fruit (imparted disposition). Participants must complete the task accurately before the baskets run out of the room on the belt: if they fail, they must repeat all trials from the beginning. The trial consists of six levels of ten tasks each. The tasks are sorted by their complexity: from high-speed tests that require few fruit movements to tests that start with a long and verbally complex command and require particular strategies to move the available fruits from one basket to another. The authors also introduced in the environment other elements such as a wastebasket, the light switch and a wall telephone, placed on the back wall, through which the subject can receive supplemental orders that in the most challenging level integrate the initial verbal command. The examiner can introduce several distracting elements to increase difficulty, generate time pressure and elicit managing strategies, such as room light fainting or progressive dimming, telephone ring and belt speed modification. Moreover, the experimenter can increase the number of sessions to execute or fix a maximum limit of “moves” that the subjects can perform to solve the trial, forcing them to follow the most efficient and quick strategy. During the trial, the subject can intervene on some additional commands, such as stopping the belt, ending the test, or freezing. For each trial, the system records many data about the subject's performance: accuracy, execution time, moves, strategical planning, and the managing steps taken to address distractors or difficulties (the high limit for the frontal patient) (Lo Priore et al., 2003).

Lo Priore and colleagues developed two versions of this V-STORE: the original immersive version (IVR-STORE) and a non-immersive version (flat screen V-STORE) (Lo Priore et al., 2003). The IVR-STORE works with IVR hardware (HMD and orientation tracking): the orientation of the subject's viewpoint and the central pointing crosshair follow real-time subjects' head movements. On the contrary, the flat screen V-STORE was programmed to work with a flat-screen monitor, and the orientation of the subject's viewpoint follows joystick input. However, in both versions, the subjects use two buttons of a joystick, one for moves in forwarding (no backward or lateral movements are possible) and the other to take or drop products. In a pilot study, Lo Priore and colleagues evaluated the sense of presence experienced by twelve young, healthy subjects in both versions (Lo Priore et al., 2003), showing a higher sense of presence perceived in immersive condition.

### Virtual Reality Platform

#### Active Brain Trainer

To work on EFs through games in multiple realistic contexts, Shochat et al. decided to implement a novel exergames platform, the Active Brain Trainer (ABT) (Intendu Ltd., Herzliya, Israel) (Shochat et al., 2017). This software adapts to the patient's behaviour in real-time and provides feedback and rewards, improving usability and compliance. This novel VR platform was designed to train specific EFs (i.e., response inhibition, sustained attention, multitasking, cognitive flexibility, working memory, planning, self-initiation and persistence, and multitasking). In the games, participants must perform several cognitive challenging tasks in real-life situations that require the combination of multiple functions simultaneously: each game focuses on one primary EF and then on a secondary one to increase transfer to real-life performance. In the VE, the authors introduced realistic situations such as interaction with people, food and transportation and a clock that shows the remaining time (minutes) to the end of the game. These funny, engaging, realistic real-life scenarios enhance daily life functioning and ensure ongoing motivation. During the game, the players see themselves within the VE through an avatar and can interact with the environment and perform the different games through natural body movements and gestures reproduced by the avatar. Finally, the high variability of contexts and actions increases gameplay richness and novelty. All ABT games are based on evidence-based paradigms, useful in training or assessing cognitive functions (e.g., CPT). The ABT games are not predetermined, but they progressively adapt in real-time to the player's performance, following both success rate and reaction time. These real-time adaptations are important to maintain high accuracy levels and to allow each player to progress in training at their own pace, adjusting the games to individual capabilities (and not vice versa) and to rehabilitation needs. Besides, the authors argued the importance of providing constant internal motivation, which would improve engagement, compliance and learning and plasticity processes. For this reason, they embedded in the program multiple levels of feedback and rewards. First, the patient receives positive audiovisual feedback immediately after a correct response, accompanied by earning virtual coins. The coins accumulate in a “coin jar,” a scoring mechanism. On the contrary, incorrect answers produce negative audiovisual feedback and the subtraction of virtual coins from the jar. Besides, players receive a cheerful sound when they reach a higher game level and see an animation that shows the advancement along an axis of levels. These elements are essential to convey to the participant a sense of progress. The level numbers presented on this axis are maintained across sessions to provide feedback within and between session progress. Finally, at the end of each game, players receive virtual gold medals and a message: they are rewarded for their progress concerning the previous session. The subjects receive one medal for playing despite achieving a lower score; 2 medals to maintain the same game level as in the last session, and 3 to pass the game level of the previous session. An example of a game is the “Bad Neighborhood” in the Food Truck Owner environment. It targets response inhibition primarily and then sustained attention, processing speed, decision-making and cognitive control. The Bad Neighborhood training game is based on Go/No Go task and CPT (Rosvold et al., 1956). Participants must perform a motor response to one stimulus class and hold a response to another class. During the game, the player represents a food truck owner who must sell food to customers. Participants must pay attention to the type of customer (“positive” or “negative”) and the several foods available on the food truck counter (CPT-like requirement). Categorization between positive and negative stimuli depends on the virtual customer's shirt color. The player must serve food to many “positive” customers (Go stimuli), avoiding thieves (No Go stimuli) to gain as much money as possible (response inhibition). Moreover, the appearance of customers with different characteristics requires speeded and automatic information processing (processing speed and decision-making) and cognitive control. Customers can approach the food truck from several positions on the counter; to serve them, the player must move his avatar (by stepping sideways or inclining his torso) to place it in front of customers. When the avatar is in the correct position, the player must execute a swipe up gesture with his arm to allow the avatar to serve the food. If the player does not serve a customer, he will turn around and walk away. After success in the primary task, other types of No-Go stimuli are introduced, such as the appearance of rotten food, which the player should avoid serving. The player earns virtual money by serving positive customers and loses money for serving negative customers or rotten food. Task parameters (e.g., probability of No-Go stimuli and a number of negative stimuli categories) are adapted gradually based on the player's success, considering both Go and NoGo trials to avoid a sudden and abrupt change in the game's difficulty. The game's overall speed is based on the reaction time in successful Go trials and is controlled through the change of presentation time of each stimulus.

Shochat et al. showed the feasibility, potential efficacy and acceptability (good satisfaction and absence of adverse effects) of this new exergames platform for the treatment at home of executive functioning in patients with ABI (Shochat et al., 2017). Interestingly, participants reported enjoyment and satisfaction from training without adverse effects, declaring interest in including it in their treatment. Indeed, subjects supported that this training allowed them to be engaged in increasingly challenging EF activities, with a high impact on their motivation and adherence (Shochat et al., 2017). These findings are crucial because patients with ABI usually stop receiving cognitive treatment when they leave the rehabilitation due to the high cost of therapy sessions and mobility to access therapy.

#### Virtual Reality Avatar Interaction Platform

Another VR platform, known as the Virtual Reality avatar interaction (VRai) platform, was designed and developed to be applied to a dual-task walking protocol to assess executive dysfunction (Robitaille et al., 2017). This platform is part of a project developing VR to rehabilitate different injuries of military personnel. VRai platform allows immersing people into varied VEs through avatars, coordinating motion capture system (MoCap), interaction and rendering system (IRS). A projection device (such as HMD) permits to present first (participant-controlled- FPA) and third-person avatars (TPA) within the specific context. The participant's full-body movements were mapped onto the FPA by the IRS. In particular, these movements are first acquired using a MoCap system. Then, these data are sent through a local network to an IRS, which applied the MoCap data to a real-time FPA representing the participant and controlled the interaction between the participant and different elements (including TPAs) of a VE. The first-person view of the VE is displayed through HMD, and the VE is updated according to the participant's head movements. The platform is designed to be functional on a broad set of VR related hardware and software for different clinical goals and populations (Robitaille et al., 2017). The environment consists of a virtual village with a central walking area of oval shape for a military patrol task. This VE was designed in-house using Softimage (Autodesk Inc., San Rafael, CA) and Blender. The participants must walk a “figure eight” path between four flags virtually hanging on a wooden. The authors included strategically placed objects (wood beams, barbed wires, and a fence) that delimited the participant's area and many buildings with windows. As part of the dual-task patrolling protocol, 2D faces can appear in different windows for set periods by opening a set of shutters. In this VE, the participant can see an FPA, developed with the open-source Makehuman 1.02 tool (www.makehuman.org) and rigged in Blender (Blender Foundation, Amsterdam, The Netherlands). Before beginning the task, the authors introduced a calibration phase in which the participant had to perform predefined body movements. The positions and orientations of the body movements (in order: segments pelvis, thorax, femurs, head, leg, feet and arms) were transmitted in real-time to the IRS to be mapped onto the FPA. To obtain these segments, twenty-nine reflective markers were placed on the participants according to the HumanRTKm model from Vicon. After that, participants must walk for patrolling VE, and their gait is observed to detect any abnormalities (e.g., hesitations). The “figure eight” shaped path started from flag one and continued in numerical order until its return to flag one. However, the path finishes at flag 2, so when the participants return to flag 1, they must continue their walking until flag 2. The participant was asked to keep a steady pace without stopping when changing direction at each flag. After the exploration of all the environment, they receive a simulation of a rifle usually used (i.e., the Colt C7A1) with a shoulder strap (this element adds specific military context), that indicates to respond to the cognitive task: with the thumb, the subjects can push on two switches located approximately where the fire control selector would be to answer to the window stimuli. Moreover, participants can walk safely or can meet some obstacles. In particular, the authors introduced three avatar conditions (TPAs) and an inanimate barrier (fence) to circumvent. TPAs with different levels of interaction are used to increase the difficulty and add more realism to the navigation task. Blender was rigged for skeletal animation with “idle” and “walk” cycles. The participants must circumvent a standing idle TPA, a walking TPA with a set, straight-line trajectory and no interaction with the participant's movement, and a walking TPA with the ability to react to the participant's movements. The two walking TPAs were positioned beyond the ends of the straight trajectories of the “figure eight” path (1-3 and 2-4 flags) and triggered to begin movement based on the participant's position along these paths. The TPA's walking speed and the direction are coordinated to the participant's velocity to meet the FPA near the centre of the patrol area. Besides, in this VE, the participant must perform a cognitive task of recognising faces in the windows (divided attention and working memory). These faces were previously declared as “hostile” or “non-hostile” and are presented at the windows for 3 s. The participant must respond to Hostile or Non-hostile as quickly as possible using the two corresponding electric switches on the rifle (up for hostile and down for non-hostile). The authors introduced two difficulty levels: the subjects must remember 2 or 4 hostiles out of 20 possible faces. Each level of facial recognition (2 vs. 4 hostiles) was presented randomized. Outcome measures involve walking data and cognitive performance. Walking based data included the ability to cross the obstacles, the mean trunk velocity and fluidity. These trunk-based dependent variables were calculated using markers on the left and right shoulders. Moreover, the cognitive performance on the recognition task involves the number of errors and reaction times in several conditions (no obstacle, fence, idle avatar, straight path avatar, interacting avatar) and dual-tasking (absence/presence of window stimuli). Overall, a preliminary study conducted on military personnel with/without mild TBI showed that the military population tolerated the VR platform, felt immersed in the VEs and enjoyed the experience, indicating that the system can be used to provide ecologically valid VE for allowing an evaluation of specific EFs (attention and navigational planning) and cognitive-motor rehabilitation in a military population (Robitaille et al., 2017). Overall, the findings showed that avatars, particularly more interactive avatars, are viewed differently and can be embedded within context-specific protocols to reveal subtle differences between two groups. Future works will have to involve greater human features (facial emotion, speech, etc.) to increase VE's ecological nature within the rehabilitation context.

### Virtual Reality Program

Other authors paid attention to the design, development and implementation of innovative VR-based tools for the rehabilitation of EFs.

#### Computer-Assisted Rehabilitation Program

In 2012, Dores et al. developed the Computer-Assisted Rehabilitation Program (CARP-VR), an innovative VR-based tool for the rehabilitation of executive functioning in patients with ABI (Dores et al., 2012). The development of this new rehabilitative instrument involved Sohlberg and Mateer's Model (Sohlberg and Mateer, 1989) and several works about VR technology and serious games (Costa, 2000; Machado et al., 2009). CARP-VR consists of two distinct VEs that simulate real-life contexts where participants must perform several activities (e.g., shopping). The tasks included in each activity have increasing complexity according to subjects' performances. The first VE, called Training Environment, represents a house, while the second one, the Rehabilitation VE, represents a supermarket. In the Training Environment, subjects can explore three scenarios, storage room, dining room and bedroom, in which they have to resolve various simple tasks of increasing complexity. Each situation requires a specific skill, respectively, recognition, sorting and problem-solving. These environments had a double aim. In the development process, it could help to decide the design, hardware, software and visualization system, allowing to evaluate the degree of user satisfaction. Moreover, it allows participants to familiarize with the VR technology and train navigation before beginning the rehabilitation program (Dores et al., 2012).

The Rehabilitation Environment is a real-time simulation of a supermarket, developed using NeoAxis Engine, in which subjects can perform any everyday tasks. It consists of two parts: an assessment phase and a rehabilitation phase. In both stages, participants must perform the same exercises, but in the first one, the tasks have an intermediate level of difficulty, while the second includes several tasks (different for each cognitive function) with varying difficulty levels. The user can decide the different levels, products, areas, and properties (e.g., price and category). Several variables are combined with increasing the complexity of the tasks in the program: list format (auditory or visual), number of items to be purchased, products' list (visible or not), number of sections and the presence or absence (Yes/No) of instructions, delayed start, repetition, error allowed, corrections, products' prices, supermarket map display, alarm, magic words (for the training of self-instruction), time limit, temporal assessment and special requirements. Each level consists of several tasks that the patients have to perform and complete successfully to progress in their rehabilitation (Dores et al., 2012). In the VE, all elements from shelves to products were modelled using Maya software; the different elements are placed according to their type: shelves and other stationery elements become static, while products are assigned to a particular interactive object. In addition to objects, the environment has specific areas to 1) to “know” if the patient visited those sections inside the supermarket.; 2) to perform particular tasks, such as taking a ticket for the line in the meat section. T Beyond the more straightforward tasks performed in a supermarket (e.g., selecting a product from a shelf and paying), other rules were included to create the levels and help patients in their rehabilitation process. For example, the therapist can add a mini-map, a shopping list (with the quantity of products to be bought), magic words, time available to complete all tasks (that includes the collection of the products and their payment), possible money (that can be exact for purchase to do, below or above) and the areas that the user must visit. Each level has a textual (and sometimes auditory) description shown at the beginning of the level, with the tasks performed. The therapist can introduce other auditory cues in the program, such as right/wrong sound or a particular sound when the patient chooses a product, not on the list. All other interactions are based on the joystick. The participants can move and interact using t a joystick by moving its principal axes to overcome the difficulties presented by patients, who have often coordination problems. By rotating the handle, patients can watch up and down. The same button can be used for all actions, such as dialogue box interaction and buying a product. The help buttons, shopping list, map, return items to the shelf are associated with keys in the joystick to avoid the use of the cursor on the screen. All purchased products are visible in the cart, so the patient does not have to remember which he has already bought. If the shopping list is available, any purchased product on the list will disappear from it, indicating that the product has been purchased. Products that are not on the list can be inserted into the cart (or not), according to the rules of each level. Moreover, the simulation ends when all products on the list have been bought or when the patient achieves the payment area (and successfully pay for the products in the cart). For example, if the patients have to purchase and pay for the products, they will see in the payment area a dialogue box with all the items in the cart, the cost of each one and the total amount, like in a real supermarket. The patient must select all products on the shopping list and have the money: if he has enough money, he must pay in cash; otherwise, he must pay by credit card. When the patient completes a level in the whole rehabilitation program, a new simulation will be presented with extra difficulty. In case of failure, depending on the settings, the patient must repeat the level until he has completed it, or the difficulty level can be lowered (Dores et al., 2012).

In the last years, several authors started to support the development of Games and 360° environments as innovative and feasible solutions for the assessment and rehabilitation of EFs.

### Virtual Reality Games

#### Virtual Reality Video Game

Pallavicini et al. explored the effectiveness in assessing EFs using a commercial VR game - Audioshield - a VR-based dance game that combines the advantages of VR and video games (Pallavicini et al., 2019). Audioshield is a dance game in which balls fly towards the player, who must follow the beat of the music to hit them successfully. This dance game incorporated cognitive engagement and physical activity, with possible benefits for EFs (e.g., inhibition of responses and working memory). Participants had to wear the HMD and used the HTC Vive's handheld controls to operate blue and red shields. Participants have to deflect the balls red, blue, or purple. Red balls must be deflected with the red shield (controlled by the right hand), while blue balls must be hit with the left hand. Moreover, purple balls require a combination of both arms. Audioshield is played with the Vive, and the physical movements are within a limited play area (4 × 4 m). During the game, the colour and the direction of the balls change continuously, requiring the user to respond correctly very quickly. After a brief explanation of the video game, participants had to perform a training phase of 2 min with Audioshield using the song “Engage” to familiarise themselves with the video game tasks and controllers. Subsequently, the individuals had to complete the song “I drop gems” while their performance was evaluated. All game lasted about 5 min. Outcome measures were: (a) the technical score (i.e., how many balls players hit - from 0 to 10.00), (b) the number of balls the player missed, and (c) the numbers of orbs the player hit. The validation study involved 38 healthy young adults and showed that the performance of VR video games correlated significantly with one at traditional neuropsychological test (TMT), suggesting that a VR game was able to measure (and treat) the same components of executive functioning (e.g., inhibition) (Pallavicini et al., 2019). Further studies will have to deepen this promising result, evaluating the efficacy of Audioshield in discriminating between clinical and healthy samples.

#### Virtual Reality Game for Executive Function Training

Shen et al. developed VR-based cognitive training for EFs rehabilitation among children with TBI (Shen et al., 2020a,b). The VR system included three VR games for training three main EFs: inhibitory control (game 1), working memory (game 2), and cognitive flexibility (game 3). Authors used the Unity game engine for developing game content, Maya 3D software for 3D modelling and animation, and Photoshop (Adobe) for 2-dimensional assets. To perform this cognitive training, the participant had to wear the VR headset to be immersed in the virtual world and interact with it by Vive controller: a “virtual hand” with which the users pressed virtual buttons to answer. Interestingly, the authors minimised the headset weight upon the child's head with TBI, skull fracture, and scalp sutures. Authors mounted the VR headset to an adjustable mechanical arm attached to a cart, reducing direct contact and weight on the head. This innovative mechanical support system allowed for using this training in sitting and reclining positions; therefore, users could experience VR in a chair or hospital bed. Interestingly, the authors chose a PC-tethered system that allowed using a separate interface for the therapist, in which they could enter user information, chose the training module, customised training by setting the number of trials and monitored VR training progress (Shen et al., 2020a,b).

At the beginning of training, a story narrative was told to participants, in which they had to “Rescue the Lubdubs”: “Lubdubs are magical creatures that live in a different world. They have been captured, and your job is to return them safely to their homes. You will play three mini-games. Our goal is to get through all the castle guards by completing each of the three games and rescuing the Lubdubs within the castle.” Game 1 was based on a classic psychological task for inhibitory control, the Spatial Stroop Task (Lu and Proctor, 1995). In this game, the user had to battle with some characters. An arrow appeared randomly to the left, right, above, or below the character during the game. The direction of the arrow may or may not be the same as its position relative to the character. At the bottom of the screen, the user could see four arrows with different directions (left, right, above, below): he had to tap on the arrow that matches the arrow that appears in the middle of the screen next to the character. In this game, the user needs to ignore the positional cue (e.g., right of the character) and respond to the actual direction of the arrow (e.g., up arrow). Game 2 is adapted from the Visual Working Memory Task (Baddeley, 2003). In this game, the user saw a locked door with different characters around the door. To unlock the door, the user needs to remember the order of characters displayed on the centre of the door. The game starts with a sequence of 2 characters and increases or reduces based on the user's responses (2 consecutive sequences correct/incorrect). Moreover, the game asked the user to recall the displayed sequence in forwarding (memory) or backward order (working memory). Finally, game three is adapted from the WCST. Participants had to send the Lubdubs back to their homes, understanding what sorting method. Specifically, they had to match the symbol on the Lubdub's stomach to one of the four symbols in front of the houses. Each choice will produce correct or incorrect feedback, and the user will need to determine the current rule based on the response. The rule changed every seven trials, although this is undisclosed to the user. In all games, outcome measures consisted of time taken to respond and accuracy of the answer. These measures allow clinicians to increase the level of difficulty of cognitive exercises, tailoring the rehabilitation according to the needs of participants.

A The pilot usability study showed that both controls and patients with TBI reported a good usability score, a high level of fun and engagement with the VR games, low levels of simulation sickness, and very light exertion due to playing the VR games. All participants could complete all three games, although patients showed a longer time to answer and a lower percentage response accuracy (Shen et al., 2020b). Future studies should expand the clinical sample and assess children's preferences by comparing VR-based and standard rehabilitation programs.

#### Exergame - Fruit Ninja

Huang and colleagues showed that the combination of immersive VR and exergames (Fruit Ninja) enhanced the feeling of presence with the potential to improve EFs in midlife and older adults after a 4-week training (Huang, 2020). In Fruit Ninja, the players used an HMD (Oculus Rift) to play the exergame and arm and hand movements to swing virtual swords to slice fruit. Data showed that immersive exergames significantly improved inhibition and task switching after the 4-week training, evaluated with traditional neuropsychological tests (Stroop Test, TMT, and Digit Span). Furthermore, a correlation appeared between the improvement in EFs and the sense of presence in the immersive experience: when participants felt immersed in the environment and perceived the possibility of moving within the environment, they improved their inhibitory control and task switching (Huang, 2020). Overall, this VR-based tool could combine the attractiveness of video games and the cognitive benefits on executive functioning.

### 360° Environment

In recent years, some authors have begun to use 360° environments (immersive photographs or videos) delivered via smartphones to present neuropsychological stimuli (Serino et al., 2017). The 360° technology can be included in the “virtuality continuum” of Milgram in which stimuli are presented in a space between real and virtual, “mixed reality,” where the extremes may co-exist, producing new experiences (Milgram and Kishino, 1994). The 360° technologies allow participants to be immersed in everyday scenarios from a first-person perspective. In this direction, Serino et al. (2017) developed a 360° version of the Picture Interpretation Test (PIT) (Rosci et al., 2005) that investigated active visual perception in patients with frontal lobe damage. PIT 360° environment consists of a present-day adaptation and small-scale colour reproduction (19 × 13) of the famous painting “Il Sorcio” (“The Mouse,” 1878, by Giacomo Favretto). The picture represents a contemporary real-world room, in which three scared girls stand on chairs and a boy is looking for something on the floor behind a cabinet. Although not visible, it is evident that there is a mouse (or a small animal). Participants undergo a visual exploration task in which they are asked to interpret what is happening in a limited time frame. This 360° environment was developed with the Ricoh Theta S Digital Camera that permits the creation of 360° spherical images with good resolution (1792 × 3584 pixels). Moreover, the Ricoh Theta S application on an iPhone 6 Plus allows a presentation of this immersive 360° experience directly on a VR headset (including mobile phone). The assessment with PIT involves two phases: “Familiarization” and “Experimentation.” Thus, the two scenes were recorded. In the familiarization phase, the scene represents a meeting room with several objects (e.g., a table, a sink with a mirror, another table with a television, two dressers and various chairs). The experimental scene was developed according to Favretto's painting “Il Sorcio.” In the same previous setting, four subjects were introduced: a boy searching for something on the floor and three frightened girls standing on chairs that watch him. Participants must sit on a swivel chair (turn on themselves) and wear the VR headset (connected to the mobile) (Serino et al., 2017).

In the familiarization phase, the examiner asks participants to keep their eyes closed until he says: “Open your eyes.” From this moment, the 3 min start. The examiner introduces the scene to participants and asks them to find some objects and answer some questions (i.e., “Let's search for the agenda. Where is the agenda?”). At the end of this phase, the examiner removes the viewer from the subject and investigates any adverse effects (e.g., dizziness, nausea). Then, participants are asked to close their eyes again and wear the viewer. The experimental session begins with the examiner's instruction, “Open your eyes.” At the same time, time registration (in seconds) and audio recording start. In this phase, participants must freely explore the scene derived from Favretto's and tell as quickly as possible what is happening (maximum time: 180 s). Time registration lasts until the participant says the word “mouse” or similar (e.g., “snake,” “roach”). After participants pronounced the correct answer, the experimenter asks: “What do you mean?” to confirm the participant's understanding of the situation. The outcome measures include 1) Correct Interpretation of scene; 2) Interpretation time (in seconds): the time between “Open your eyes” and correct interpretation (max time allowed is 180). If the subjects fail to interpret the scene, the examiner gives 180 s as interpretation time (as suggested by Rosci et al.); 3) The number of Scene Elements: The sum of the scene elements verbalised during the scene's interpretation. After developing PIT 360°, Serino et al. evaluated its efficacy in discriminating patients with PD and healthy controls. Results showed that both traditional neuropsychological assessment and PIT 360° revealed different performances in PD patients compared to controls: patients took longer to provide a correct interpretation of the scene proposed, gave significantly more detailed descriptions of the scene and appeared more prone to distractor interference. Thus, patients showed more difficulties in focusing on the most critical components for a correct interpretation of the scene (Serino et al., 2017). These findings align with Luria's view, suggesting that this test can capture deficits in active visual perception. In the following study, Realdon and co-workers obtained similar results in detecting executive impairments in MS (Realdon et al., 2019). Interestingly, PIT 360° allowed differentiating patients with MS and controls, although the global cognitive level and standard neuropsychological tests of executive functioning were still in a non-pathological range (Serino et al., 2017). Thus, these findings suggested that PIT 360° was an ecological tool that has been highly sensitive for detecting deficits of EFs since the early clinical stage of MS.

Recently, Borgnis et al. developed the “Executive-functions Innovative Tool 360°” (EXIT 360**°**), an innovative, enjoyable, and ecological tool for a multidimensional and multicomponent evaluation of executive dysfunctions (Borgnis et al., 2021a). EXIT 360° was born to provide a quick, complete and integrated EFs evaluation through an original task for EFs delivered via a comfortable mobile-powered VR headset possibly combined with an eye tracker and electroencephalogram. EXIT 360° allows participants to engage in a “game for health,” delivered via smartphones, in which they have to perform several everyday subtasks in five 360° daily environments (i.e., kitchen, two bedrooms, living room, and landing). Specifically, participants aim to leave the domestic setting in the shortest possible time, overcoming seven subtasks of increasing complexity designed to tap and evaluate different components of executive functioning (e.g., planning, decision-making, problem-solving, attention, visual-searching, and working memory). The examiner accompanies and guides the participants along the entire path, providing the subtasks' instructions, collecting all the subjects' verbal answers, and managing the transition from one level to another of greater complexity. EXIT 360° appears as a promising tool usable in evaluating several clinical populations that show various executive dysfunctions. The potential clinical applications of this innovative tool could radically transform patients' and clinicians' assessment experience. On one side, it enriches the assessment of EFs by integrating verbal responses, reaction times, and physiological data (eye movements and brain activation), allowing the clinician to obtain, in real-time and simultaneously, a wide range of information about executive dysfunction and its impact in real life. On the other side, EXIT 360° involves patients in a task that can be experienced as a game, with high levels of engagement and decreased anxiety levels. Preliminary studies have shown promising results in terms of usability, involving healthy control subjects (i.e., EXIT 360° appeared usable and easy-to-learn tool) and convergent validity (i.e., EXIT 360° was able to evaluate executive functions in healthy controls) (Borgnis et al., 2021b). Further studies will have to assess the usability of EXIT 360° in clinical populations and its efficacy in discriminating between healthy controls and clinical populations.

## Conclusion

This review conceives to provide a detailed description of all innovative VR-based instruments currently available to assess and rehabilitate EFs, a complex construct involving several higher-order cognitive and behavioral skills which play a key role in daily life and independent functioning (e.g., preparing meals, managing money, shopping) (Josman et al., 2009; Diamond, 2013). Due to this crucial role in daily functioning, identifying early strategies functional to the evaluation and rehabilitation of EFs in real-life scenarios appears necessary to minimize the effects of executive impairments, improving everyday functioning and quality of life (Levine et al., 2007). Different instruments have been developed in real-life contexts with the advantage of obtaining a more accurate estimate of the patient's executive deficits than within laboratory conditions (Rand et al., 2009). However, numerous difficulties have been highlighted in the literature about the administration of tests or training in real-life scenarios, such as long times, high economic costs, patients' safety risk, poor controllability of experimental condition or applicability with patients with motor deficits (Rand et al., 2009; Raspelli et al., 2009; Parsons, 2015).

All these problems have paved the way to use technological tools and, specifically VR, to evaluate and rehabilitate EFs in real-life ecologically (Bohil et al., 2011; Parsons et al., 2011; Parsons, 2015), with rigorous control over the key variables (Campbell et al., 2009). Indeed, over the years, VR-based tools appeared a promising solution in neuropsychological assessment and rehabilitation, able to early detect and treat everyday cognitive impairments, minimizing the impact on daily functioning (Negu et al., 2016).

This systematic review involved 100 studies that described the primary VR-based tools for assessing or rehabilitating executive functioning. In the last decade (2010-2021), the studies on VR and executive functioning have triplicated compared to those in the previous decade (1998–2009). Specifically, 23 studies were carried out between 1998 and 2009 (first phase) and 81 between 2010 and 2021 (second phase). The spread of VR proceeded in parallel with developing hardware and software more reliable, cheap, and acceptable in size (Bohil et al., 2011). This spread, along with the awareness of the importance of an ecological instrument for the evaluation and rehabilitation of EFs (Campbell et al., 2009; Parsons et al., 2011; Parsons, 2015), have contributed to a revaluation of the traditional tools (Chaytor et al., 2006) and increased research on VR instruments (Parsons, 2015).

To date, most available tools have focused on the evaluation of executive functionality than rehabilitation, showing significant variability in terms of implemented settings, stimuli, and tasks. We have provided a detailed description of 30 VR-based assessment tools, specifically for evaluating executive dysfunctions.

Most reviewed studies involved different numerous computer-simulated everyday scenarios (e.g., supermarket, kitchen) in which subjects could interact dynamically with 3D objects in real-time, “like in real life” (Pratt et al., 1995; Climent et al., 2010). Our work shows that virtual supermarket is the most used VE (e.g., VMET), followed by VC and virtual Kitchen. Over the years, the researchers implemented further VEs involving specific real-life contexts to improve the ecological validity of tests, such as offices, city, library, or buildings. In these everyday environments, participants must perform several tasks involving complex real-life situations (e.g., shopping and cooking) that require subjects the use of several EFs (Nir-Hadad et al., 2017), mirroring the cognitive demands of daily functioning (Chaytor and Schmitter-Edgecombe, 2003). The high flexibility and programmability of VR are a critical component in the evaluation tool since they guarantee the controlled and precise presentation of a large variety of stimuli and distractions/stressors that patients may meet in their everyday life (Armstrong et al., 2013). From our revision, shopping has been selected as the best activity since it includes several tasks/actions that involve many EFs (planning, multitasking, problem-solving, set-shifting), showing sensitivity not only to clinical conditions (healthy control subjects vs. clinical population) but also to ageing (young vs. older healthy subjects). Indeed, a promising characteristic emerged in the works reviewed regards the ability of some VR-based tools to assess executive functionality controlling for main demographic or clinical features, such as age, education or global cognitive functioning (Renison et al., 2012). Finally, many innovative VR-based tools were based on existing ecological tests, for example, VMET or virtual version of Library Task. The original version of this test appeared to be able to overcome the ecological issue of traditional paper-and-pencil tests, providing clinicians the opportunity to evaluate executive functioning in real-life scenarios but showed the numerous limitations described above. In this framework, the virtual versions of these ecological tests allowed to overcome all these difficulties. Similarly, several authors have successfully proposed virtual versions of the traditional neuropsychological paper and pencil tests for executive functioning (e.g., ToL) that allowed to go beyond the overt ecological issues. Finally, in the last years, several authors started to support the development of Games and 360° environments as innovative and feasible solutions for the assessment and rehabilitation of EFs. For example, SG is a digital application that can be considered a promising non-pharmacological tool to evaluate and treat patients' functional impairments (Robert et al., 2014).

In addition to the complete description of tools, we have focused on their psychometric properties, particularly construct validity, discriminant validity, usability and test re-test reliability. Overall, most of these VR-based assessment instruments (77%) have good construct validity, showing significant correlations between the primary outcome measures and the score of existing standardized paper-and-pencil tests for executive functioning, particularly TMT and Stroop Test. Despite these promising results, to date, the works have shown a good discriminant validity only for half of the developed instruments. Among the tool that appeared efficacy in discriminating between populations, usually between healthy controls and pathological conditions, the studies have converged in supporting the feasibility and effectiveness of VR-based tools in the ecologically valid evaluation of executive functionality in psychiatric (i.e., OCD and Schizophrenia) and neurologic (acute, neurodegenerative, and neurodevelopment) populations. Interestingly, the VR-based instruments appeared able to early detect executive dysfunctions before the onset of cognitive dysfunction, for example, in non-demented PD or HD premanifest (Cipresso et al., 2014). These results appeared important since an increasing number of longitudinal studies suggested that early executive dysfunction is predictive of the PD conversion in PD with dementia (Azuma et al., 2003; Janvin et al., 2005). Thus, the early identification of executive deficits could permit identifying patients at risk to develop dementia, providing early neurorehabilitation interventions (Cipresso et al., 2014; Serino et al., 2014). Similarly, identifying and quantifying subtle disease-related alterations in individuals who carry the abnormal gene but do not yet meet the criteria for a clinical diagnosis of HD provides a new opportunity for interventions to prevent or delay the onset of symptoms (Weir et al., 2011). It should be noted that the lack of discriminant validity constitutes a significant limitation in the use of these tools since the absence of information on diagnostic specificity and sensitivity in clinical populations makes impossible to introduce them into clinical practice. Moreover, this review highlighted the lack of studies on two other critical components for an instrument exploitable in a clinical setting: usability and test-retest reliability. Only four studies have focused on usability evaluation, showing that tools appeared usable, easy-to-learn, challenging and engaging, and free from significant side effects (Aubin et al., 2018; Borgnis et al., 2021b). Over the years, several studies have shown the crucial role of assessing usability and user experience in developing VR-based tools (Pedroli et al., 2013, 2019; Sauer et al., 2020; Tuena et al., 2020). The usability assessment allows understanding the “degree to which a subject is able to use a system to achieve specific goals effectively, efficiently, and satisfactorily within a well-defined context of use” (Iso, 1998). Overall, the usability and user experience evaluation allow for understanding any difficulties that could affect subjects' performance, including adverse effects or technological expertise. Previous evidence showed that cyber sickness could lead to unpleasant experiences for the users, affecting their performance and significantly decreasing the test results' validity (Armstrong et al., 2013). For example, in the TBI population, headaches are a common symptom; therefore, a tool that could exacerbate these symptoms would impact the performance, decreasing the test results' validity. In this framework, the crucial role of these variables clearly appears; future studies will necessarily have to investigate these aspects involving both healthy subjects and several clinical populations. As regards the last critical components, to date, only one work has focused on test-retest reliability (Plotnik et al., 2021), showing interesting results. The concept of test-retest reliability assumes a critical role in the clinical setting in which clinicians must longitudinally monitor the patients, for example, during a rehabilitative path. Further studies will have to be conducted to evaluate test-rest reliability, measuring the consistency of results or repeating the same test on the same sample at a different point in time or comparing a test with its parallel forms.

Regarding the rehabilitation of EFs, studies showed that VR-based instruments could be considered a promising solution in treating several components of this complex construct due to specific characteristics of VR-based training. Firstly, the VE adapts to the patient's performance in real-time. Therefore, clinicians can not only monitor the rehabilitation in real-time but also tailor it according to individual needs and progress, improving usability and compliance (Lo Priore et al., 2003). The theme of flexibility and programmability of VR also assumes a critical role in rehabilitating executive dysfunction. In fact, the clinician can tailor the rehabilitation, introducing real distractors and stressors that patients may meet in their everyday lives. Moreover, they could provide cueing stimuli to patients to help them in compensatory strategies to improve everyday functional behaviour (Rizzo et al., 2001). Moreover, VEs can also be programmed to treat patients with reduced sensory and motor skills. Indeed, VR allows administering stimuli and instructions through different modalities (visual, auditory, tactile), adapted to the patients' possible sensory deficits (Parsons et al., 2008). In addition, the reviewed studies have shown the ability of VR to provide immediate feedback on performance and rewards, allowing participants to optimise the performance (Shochat et al., 2017).

Furthermore, the enjoyment and attractiveness of VR allowed increasing motivation and participation of participants, leading to extensive training and greater cognitive improvement. This evidence is in line with literature that considers VR as a promising tool to improve rehabilitation since it allows the provision of meaningful, versatile and individualized tasks that can enhance patients' motivation, enjoyment and engagement during training (Hayre et al., 2020), overcoming scarce compliance of patients with cognitive dysfunctions about the traditional rehabilitating program, usually repetitive and not stimulating (Castelnuovo et al., 2003; Rand et al., 2009). In addition, since VR simulation tasks were more similar to daily activities than those used during conventional therapy (Liao et al., 2019), the participants were able to transfer rehabilitation results from the VR treatment to function in the real world. Finally, VR allows patients to perform exercises in their homes' comfort and safety at a distance. This result has been relevant since it makes it possible to overcome two crucial clinical issues: long waiting lists of health services and difficulties in moving patients between their homes and health services.

Despite all these promising results, several authors have underlined in their studies the presence of some disadvantages in using a VR-based tool to assess and rehabilitate executive functioning. Firstly, the level of familiarity of users with technology appeared critical, above all older adults, since poor performance in the test could be due to insufficient knowledge of how VR works (Parsons and Phillips, 2016). However, to overcome this problem, the clinician could propose subjects the training with the tool before the real test (a familiarization phase) to maximize familiarity with the technological platform (Parsons and Phillips, 2016). Moreover, the development of VR-based tools with complex VEs and tasks requires numerous specialized technological skills and high costs (Parsons, 2015). However, in the last years, the diffusion of VR has proceeded in parallel with the development of devices, hardware and software that are more reliable, economical and acceptable in terms of size. Finally, studies reviewed showed the lack of a control group with placebo or no treatment (Liao et al., 2019) and the small samples recruited (among 20 and 50 participants), although the experimental groups were well-matched for the main sociodemographic characteristics. Therefore, the following studies will have to expand the sample and introduce another treatment to confirm these promising results.

Overall, this review has shown that clinicians can consider VR an innovative and valuable solution to identify novel strategies for evaluating and rehabilitating EFs in real-life scenarios, able to early detect executive impairments and minimize their effects, improving everyday functioning.

## Data Availability Statement

The original contributions presented in the study are included in the article/supplementary files, further inquiries can be directed to the corresponding author/s.

## Author Contributions

FBo and PC independently conducted the data extraction. PC, LU, and GR supervised the sections of methods and Virtual Reality Tool. EP and FR supervised the introduction. FBa supervised the conclusion. FBo wrote the manuscript under the final supervision of FBa, JO, and PC. All authors have read and agreed to the published version of the manuscript.

## Funding

This study was funded by the Italian Ministry of Health.

## Conflict of Interest

The authors declare that the research was conducted in the absence of any commercial or financial relationships that could be construed as a potential conflict of interest.

## Publisher's Note

All claims expressed in this article are solely those of the authors and do not necessarily represent those of their affiliated organizations, or those of the publisher, the editors and the reviewers. Any product that may be evaluated in this article, or claim that may be made by its manufacturer, is not guaranteed or endorsed by the publisher.
