# Corrigendum: Available virtual reality-based tools for executive functions: A systematic review

**DOI:** 10.3389/fpsyg.2022.995038

**Published:** 2022-08-17

**Authors:** Francesca Borgnis, Francesca Baglio, Elisa Pedroli, Federica Rossetto, Lidia Uccellatore, Jorge Alexandre Gaspar Oliveira, Giuseppe Riva, Pietro Cipresso

**Affiliations:** ^1^IRCCS Fondazione Don Carlo Gnocchi ONLUS, Milan, Italy; ^2^Department of Psychology, Catholic University of the Sacred Heart, Milan, Italy; ^3^Applied Technology for Neuro-Psychology Lab, Istituto Auxologico Italiano, Istituto di Ricovero e Cura a Carattere Scientifico, Milan, Italy; ^4^Faculty of Psychology, eCampus University, Milan, Italy; ^5^Universidade Lusófona de Humanidades e Tecnologias, Lisboa, Portugal; ^6^Department of Psychology, University of Turin, Turin, Italy

**Keywords:** executive functions, Virtual Reality, psychometric assessment, rehabilitation, virtual environments

In the published article, the Funding statement was missing. The correct Funding statement appears below:

## Funding

This study was funded by the Italian Ministry of Health.

The authors apologize for this error and state that this does not change the scientific conclusions of the article in any way. The original article has been updated.

## Publisher's note

All claims expressed in this article are solely those of the authors and do not necessarily represent those of their affiliated organizations, or those of the publisher, the editors and the reviewers. Any product that may be evaluated in this article, or claim that may be made by its manufacturer, is not guaranteed or endorsed by the publisher.

